# The Spreading and Effects of Human Recombinant α-Synuclein Preformed Fibrils in the Cerebrospinal Fluid of Mice

**DOI:** 10.1523/ENEURO.0024-23.2024

**Published:** 2024-03-01

**Authors:** Charysse Vandendriessche, Arnout Bruggeman, Joyce Foroozandeh, Lien Van Hoecke, Pieter Dujardin, Junhua Xie, Griet Van Imschoot, Elien Van Wonterghem, Jonas Castelein, Cristiano Lucci, Lies De Groef, Roosmarijn E. Vandenbroucke

**Affiliations:** ^1^VIB Center for Inflammation Research, VIB, 9000, Ghent, Belgium; ^2^Department of Biomedical Molecular Biology, Ghent University, 9000, Ghent, Belgium; ^3^Department of Neurology, Ghent University Hospital, 9000, Ghent, Belgium; ^4^VIB Center for Brain & Disease Research, VIB, 3000, Leuven, Belgium; ^5^Department of Neurosciences, Brain Institute KU Leuven, 3000, Leuven, Belgium; ^6^Cellular Communication and Neurodegeneration Research Group, Department of Biology, Leuven Brain Institute, KU Leuven, 3000, Leuven, Belgium

**Keywords:** α-synuclein, blood–cerebrospinal fluid barrier, cerebrospinal fluid, choroid plexus, Parkinson's disease, preformed fibrils

## Abstract

Parkinson's disease (PD) patients harbor seeding-competent α-synuclein (α-syn) in their cerebrospinal fluid (CSF), which is mainly produced by the choroid plexus (ChP). Nonetheless, little is known about the role of the CSF and the ChP in PD pathogenesis. To address this question, we used an intracerebroventricular (icv) injection mouse model to assess CSF α-syn spreading and its short- and long-term consequences on the brain. Hereby, we made use of seeding-competent, recombinant α-syn preformed fibrils (PFF) that are known to induce aggregation and subsequent spreading of endogenous α-syn in stereotactic tissue injection models. Here, we show that icv-injected PFF, but not monomers (Mono), are rapidly removed from the CSF by interaction with the ChP. Additionally, shortly after icv injection both Mono and PFF were detected in the olfactory bulb and striatum. This spreading was associated with increased inflammation and complement activation in these tissues as well as leakage of the blood–CSF barrier. Despite these effects, a single icv injection of PFF didn't induce a decline in motor function. In contrast, daily icv injections over the course of 5 days resulted in deteriorated grip strength and formation of phosphorylated α-syn inclusions in the brain 2 months later, whereas dopaminergic neuron levels were not affected. These results point toward an important clearance function of the CSF and the ChP, which could mediate removal of PFF from the brain, whereby chronic exposure to PFF in the CSF may negatively impact blood–CSF barrier functionality and PD pathology.

## Significance Statement

Although it is well known that seeding-competent α-synuclein (α-syn) is present in cerebrospinal fluid (CSF) of Parkinson's disease (PD) patients, the CSF spreading of preformed fibrils (PFF) and the following short- and long-term consequences on the brain have not yet been investigated. Here, we show that repeated PFF administration in CSF of wild-type mice results in PD-like pathology 2 months later, underlining the pathologic relevance of the presence of seeding-competent α-syn PFF in the CSF. Furthermore, we identify the choroid plexus (ChP), an often-neglected brain region, as a potentially important player in PD pathology due to its capability to interact with CSF α-syn.

## Introduction

Parkinson's disease (PD) is the second most common neurodegenerative disorder, affecting up to 2–3% of the population ≥65 years of age (Poewe et al., 2017). One of the pathological hallmarks is the misfolding, aggregation, and widespread accumulation of the protein α-synuclein (α-syn). These α-syn aggregates build up in neurons and form the typical intracellular Lewy bodies. Intriguingly, the spreading of aggregated α-syn throughout the brain occurs via a predictable pattern called Braak stages. In the early Braak stages of PD, α-syn aggregates can only be found in either the olfactory bulb (OB) or the lower brainstem from where they start propagating throughout the entire brain (Braak et al., 2003). More recently, it has been hypothesized that PD comprises a brain-first and a body-first subtype consisting of different spreading patterns. Hereby, it is proposed that the initial α-syn pathology appears in the central nervous system (CNS; more specifically the amygdala or connected structures and in some cases the OB) before descending to the peripheral nervous system, whereas the reversed pattern is described in the body-first subtype (i.e., peripheral nervous system prior to CNS; Horsager et al., 2020). The α-syn spreading is thought to occur in a prion-like fashion from diseased to healthy neurons, whereby misfolded α-syn from the donor neuron serves as a seed to induce aggregation of α-syn in the acceptor neuron (Uemura et al., 2020). Based on this concept, a model of PD that makes use of α-syn preformed fibrils (PFF) derived from recombinant human or murine monomeric α-syn protein was developed (Polinski et al., 2018). Using this model, it was shown that a single injection of PFF in a specific region of wild-type (WT) rodent brain (e.g., striatum, substantia nigra, OB) seeds the misfolding, aggregation, and fibrillar deposition of endogenous monomeric α-syn (Luk et al., 2012; Rey et al., 2016; Abdelmotilib et al., 2017). This occurs in a progressive prion-like spreading pattern along anatomically connected neuronal networks from the site of injection throughout the brain and is associated with a gradual development of degeneration of nigrostriatal dopaminergic neurons and motor deficits (Polinski et al., 2018).

Ongoing pathological changes in the brain are known to be reflected in the cerebrospinal fluid (CSF), making this fluid that flows inside the brain ventricles and the subarachnoid space (SAS) an ideal source for diagnostic markers. Indeed, seed amplification assays (SAA) show the presence of misfolded α-syn seeds with self-aggregating properties in CSF samples from synucleinopathy patients, whereas no seeding activity could be detected in nonsynucleinopathy controls (Fairfoul et al., 2016; Shahnawaz et al., 2016; Groveman et al., 2018; Paciotti et al., 2018; Rumund et al., 2019; Rossi et al., 2020; Russo et al., 2021). Strikingly, a large-scale cross-sectional study showed that CSF α-syn SAA can differentiate PD patients from healthy controls with high sensitivity and specificity (Siderowf et al., 2023). Moreover, the assay can detect prodromal individuals before diagnosis, indicating that the presence of CSF α-syn seeds might be an early indicator of synucleinopathy (Siderowf et al., 2023). Despite the knowledge of the (early) presence of seeding-competent α-syn in the CSF, up until now, the CSF spreading of PFF and the following consequences have not been investigated. To address this knowledge gap, we used an intracerebroventricular (icv) injection model to introduce α-syn PFF directly into the CSF of WT mice, followed by an analysis of the α-syn spreading and its short- and long-term consequences on the brain.

The majority of CSF is secreted by the choroid plexus (ChP), a highly vascularized structure hanging inside the brain ventricles. Morphologically, the ChP consists of a single layer of tightly interconnected ChP epithelial cells that form a barrier between CSF and blood flowing inside leaky capillaries supplying the ChP stroma (Vandenbroucke, 2016). Together with other brain barriers such as the blood–brain barrier and arachnoid barrier, the blood–CSF barrier protects the brain against external insults (e.g., toxins, infectious agents, and peripheral blood fluctuations) and assures a balanced and well-controlled environment in the CNS (Engelhardt et al., 2017). It is well known that aging—the biggest risk factor for the development of PD—affects the morphology and functionality of the blood–CSF barrier leading to a compromised barrier integrity and reduced CSF turnover, among others (Gorlé et al., 2016; Vandenbroucke, 2016). Of note, alterations in CSF drainage and ChP transporter function are believed to contribute to Alzheimer's disease (AD) pathology by affecting the clearance of amyloid beta (Aβ; Serot et al., 2012), but it is currently unknown whether similar mechanisms apply to PD. Nonetheless, although little is known about the functionality of the blood–CSF barrier in PD, the available data point toward its potential importance.

Remarkably, in early-stage PD patients, an increase in ChP volume as obtained from MRI images was associated with a decrease in striatal dopamine transporter availability and more severe baseline motor deficits, as well as with a higher risk of developing freezing of gait and a more rapid increase in dopaminergic medication over time (Jeong et al., 2023). Furthermore, α-syn immunoreactivity is present in human ChP tissue derived from patients with synucleinopathies including PD, although the signal is variable (Mollenhauer et al., 2012). This suggests that the ChP might take up α-syn that is present in the CSF, thereby representing a potential clearance mechanism for α-syn removal from the brain. Transwell experiments using immortalized ChP epithelial cells showed that ChP epithelial cells are capable of taking up and transporting exogenously added α-syn from the inner compartment (resembling the CSF side) to the outer compartment (resembling the blood side) and vice versa (Bates et al., 2015). Additionally, an increased albumin CSF-to-serum ratio suggestive of increased blood–CSF barrier leakage has been described in PD patients with advanced disease (Pisani et al., 2012), but not in early-phase PD patients (Haussermann et al., 2001; Pisani et al., 2012). Together, these data suggest that the ChP might play a role in clearing α-syn from the brain and that the ChP functionality could be affected during PD, similar to what is described for AD.

Previously, it has been reported that the blood–CSF barrier is affected in mouse models of peripheral inflammation (Balusu et al., 2016; Gorlé et al., 2018; Xie et al., 2021), in Niemann–Pick type C disease (Hoecke et al., 2021), and in AD (Brkic et al., 2015; Steeland et al., 2018; Vandendriessche et al., 2021; Xie et al., 2021). In the present study, we assessed the functionality of the blood–CSF barrier and the role of the CSF in the spreading of α-syn PFF—two understudied topics in the PD field—using an icv injection model. This revealed that the PFF interact with the ChP, OB, and striatum, which is associated with increased inflammation and complement activation in these tissues. Additionally, the presence of PFF in the CSF induced leakage of the blood–CSF barrier. However, despite these acute effects, a single icv injection of PFF didn't induce a motor function phenotype in the long term. In contrast, 5 repeated daily icv injections resulted in PD-like pathology 2 months after injection as evidenced by the formation of phosphorylated α-syn inclusions in the brain as well as a deterioration in grip strength.

## Materials and Methods

### Mice

For short-term experiments, female C57BL/6J mice housed in a specific pathogen-free animal facility were used. The mice were between 9 and 12 weeks of age at the time of the experiments. For long-term follow-up experiments, female C57BL/6J mice (injected at 12 weeks of age) were accommodated in the dedicated motor function procedure room in a conventional animal facility. In both facilities, mice were housed in groups of 4–6 per cage with *ad libitum* access to food and water and a 14 h light/10 h dark cycle.

### Expression and purification of recombinant human α-syn

Human (NM_000345) recombinant full-length monomeric α-syn (α-syn Mono) protein was expressed using the ampicillin-resistant inducible pRK172 vector system and purified from BL21-CodonPlus (DE3)-RIL competent *Escherichia coli* cells (230245; Life Technologies) as previously described (Volpicelli-Daley et al., 2014). Briefly, the bacteria were harvested by centrifugation at 6,000 × *g* for 10 min at 4°C after overnight (ON) incubation at 37°C while shaking. The bacterial pellet was resuspended in high-salt buffer [750 mM NaCl, 10 mM Tris HCl (pH 7.6), and 1 mM EDTA] with protease inhibitors (Roche) and 1 mM PMSF (Roche). Subsequently, the pellet was lysed by sonicating with a 1/8 in probe tip for 5 min (30 s pulse on, 30 s pulse off) at 60% amplitude (VCX 1500 HV, Vibra-Cell Sonics) and boiling for 15 min. After centrifugation at 6,000 × *g* for 20 min, the supernatant was subjected to serial purification steps using a Superdex 200 column (GE Healthcare Life Sciences) and a HiTrap Q HP anion exchange column (GE Healthcare Life Sciences). Purified α-syn was subjected to endotoxin removal (LPS content < 5 EU/mg) using ActiClean Etox (Sterogene Bioseparations). Protein concentration was measured using the Pierce BCA protein assay (Thermo Fisher Scientific) followed by a concentration of up to ∼30 mg/ml. Aliquots of α-syn Mono were stored at −80°C until further use.

### Preparation of recombinant human α-syn PFF

α-syn PFF were prepared and validated following the current recommendations in the field (Polinski et al., 2018). Prior to the generation of α-syn PFF, an aliquot of α-syn Mono was gradually thawed on ice followed by ultracentrifugation at 100,000 × *g* for 45 min at 4°C to pellet any aggregated material. Subsequently, the supernatant was used to generate α-syn PFF. α-syn Mono were diluted in sterile Dulbecco's phosphate buffered saline (Invitrogen Life Technologies) to a final volume of 200 µl with a concentration of 5 mg/ml in sterile low-adhesion tubes (Biozym Scientific). The tubes were placed in an orbital mixer to shake for 7 d at 37°C whereafter the solution appeared turbid. The α-syn PFF were stored at −80°C after aliquoting and gradual freezing on dry ice. Immediately before use, freshly thawed α-syn PFF were diluted in sterile PBS in a final volume of 100 µl with a concentration of 1 mg/ml in sterile low-adhesion tubes. Next, the α-syn PFF were sonicated with a 1/8 in probe tip for 60 s (four cycles with 2 min intervals of 15 s pulse on and 15 s pulse off) at 30% amplitude (Q500, QSonica).

### Validation of PFF formation and seeding capacity via thioflavin T assay, sedimentation assay, transmission electron microscopy, and in vitro seeding experiments

To validate the β-sheet conformation of the fibrils via the thioflavin T (ThT) assay, we added 100 µl reaction mix consisting of 0.05 mg/ml recombinant human α-syn Mono or unsonicated PFF and 20 µM ThT in PBS to a 96-well plate (Sigma-Aldrich). The ThT signal in arbitrary units (AU) was determined by measuring the fluorescence (*λ*_ex_/*λ*_em _= 450/485 nm) using the EnVision multilabel plate reader (PerkinElmer) at 37°C. PFF seeding capacity was validated using a 100 µl reaction mix consisting of 0.05 mg/ml recombinant human α-syn Mono, 1 mg/ml unsonicated or sonicated PFF, and 20 µM ThT in PBS was added to a 96-well plate (Sigma-Aldrich). The ThT signal was determined as described above during 24 h at 37°C with 10 min intervals preceded by a short shaking step at 800 rpm before every measurement.

To validate fibril aggregate formation via the sedimentation assay, we diluted recombinant human α-syn Mono or unsonicated PFF in PBS to a final concentration of 0.5 mg/ml and centrifugated at 55,000 rpm for 1 h at room temperature (RT). The supernatant (100 µl) was transferred to a new tube, whereas the pellet was resuspended in 100 µl PBS. Both the supernatant and the pellet were mixed with sample buffer [0.35 M Tris HCl pH 6.8, 10% sodium dodecyl sulfate (SDS), 35% glycerol, 5% β-mercaptoethanol, 0.5% bromophenol blue] and separated on a 15% SDS-PAGE gel. Next, Coomassie staining was performed.

To visualize fibril formation via transmission electron microscopy (TEM), we spotted a 20 µl sample (0.125 mg/ml Mono, sonicated PFF or unsonicated PFF) on a parafilm sheet. Formvar/C-coated hexagonal copper grids (EMS G200H-Cu) were glow discharged for 10 s and then placed on top of the droplet for 1 min with the coated side of the grid down. The grids were washed five times in droplets of Milli-Q water, stained with 1% (w/v) uranyl acetate for 10 s, and air-dried for 24 h before imaging. Visualization of the samples was done using a JEM 1400 plus transmission electron microscope (JEOL) operating at 80 kV. The length of the PFF was measured in Fiji (ImageJ).

To validate the in vitro seeding capacity of the fibrils, we prepared primary hippocampal neuron cultures from C57/BL6 mouse embryos at the E16.5 stage of development, as previously described with minor modifications (Lucci et al., 2020). Hippocampal tissue was dissected and further digested in Hanks balanced salt solution (Ca^2+^- and Mg^2+^-free; Invitrogen) with 1 mg/ml trypsin and 5 mg/ml DNAse I (Sigma-Aldrich) at 37°C for 30 min. Following the addition of 0.05% (v/v) soybean trypsin inhibitor (Sigma-Aldrich), the tissue was mechanically dissociated in neurobasal media (Invitrogen) supplemented with (0.2 mM) 1× GlutaMAX (Invitrogen) and 2% B-27 supplement (Invitrogen). Dissociated neurons were resuspended in supplemented neurobasal media and plated onto 50 µg/ml poly-ʟ-ornithine (Sigma-Aldrich)-coated 12 mm round glass coverslips at a density of 20,000–40,000 cells/cm^2^ in 24-well plates (Thermo Fisher Scientific). During the whole experiment, 50% of the culture medium was refreshed once a week. At 7 d after plating, the cells were stimulated with 0.001 mg/ml Mono or sonicated PFF as described previously (Volpicelli-Daley et al., 2014), and immunostaining was performed 11 d later. Hippocampal neurons were fixed with 4% paraformaldehyde (Thermo Fisher Scientific)/4% sucrose/1% Triton X-100 in PBS for 15 min at RT, rinsed with PBS, permeabilized in PBS/Triton X-100 (1× PBS, 0.2% Triton X-100; Sigma-Aldrich), and blocked with 3% bovine serum albumin (BSA) in PBS (BSA; Sigma-Aldrich). Next, the primary antibodies anti-pS129 α-syn (1:1,000; SMC-600; StressMarq) and anti-β-III tubulin (1:500; NB100-1612; Novus Biologicals) were added for 2 h at RT. Following wash steps, the secondary antibodies goat anti-chicken AlexaFluor 488 and goat anti-rabbit DyLight 633 were diluted 1:500 and 1:1,000 in PBS, respectively, and incubated for 2 h at RT. Finally, after rinsing with PBS, the cells were counterstained with Hoechst (1 µg/ml) and mounted using the antifading mounting medium Mowiol (10%, Sigma-Aldrich). Imaging was performed using a Leica Stellaris 8 confocal microscope.

### Intracerebroventricular injection

The technical procedure of the icv injections was performed as described before (Brkic et al., 2015; Steeland et al., 2018; Vandendriessche et al., 2021). In short, mice were anesthetized with isoflurane and mounted on a stereotactic frame. A constant body temperature of 37°C was maintained using a heating pad. Injection coordinates were measured relative to the bregma intersection (anteroposterior −0.7 mm, mediolateral +1.0 mm, dorsoventral −2.0 mm) and were determined using the Franklin and Paxinos mouse brain atlas. By using a Hamilton needle, 5 µl of either PBS, α-syn Mono (1 µg/µl) or α-syn PFF (1 µg/µl), were injected into the left lateral ventricle. As such, we injected 5 µg of α-syn protein, which is based on a previous study (Luk et al., 2012). For the long-term follow-up experiment, the mice were injected for 5 consecutive days at the same injection site. α-Syn Mono were kept on ice and α-syn PFF were stored at RT during the experimental procedures. α-Syn PFF were used within 4 h after sonication. The subsequent sampling was performed at 1, 4, 6, 8, and 24 h after the icv injections.

### CSF isolation

CSF was collected using the cisterna magna puncture method as described before (Liu and Duff, 2008; Vandendriessche et al., 2021). Briefly, capillaries to isolate CSF were made from borosilicate glass capillary tubes (B100-75-15; Sutter Instrument Company) using the Sutter P-87 flaming micropipette puller (pressure, 330 Pa; heat index, 300). Just before CSF isolation, mice were anesthetized with 200 µl ketamine/xylazine (100 mg/kg, ketamine; 20, mg/kg xylazine). An incision was made inferior to the occiput and disinfected with 70% ethanol. The dura mater was exposed by separating the muscle tissue on the dorsal side of the skull. Next, the animal was mounted at an angle of 135°, and CSF was collected by puncturing the dura mater of the cisterna magna using the capillary needle.

### Blood–CSF barrier permeability

Blood–CSF barrier permeability was determined as described previously (Vandenbroucke et al., 2012; Brkic et al., 2015; Steeland et al., 2018). In brief, mice were intravenously (iv) injected with 250 mg/kg FITC-labeled dextran (4 kDa, Sigma-Aldrich) 15 min before CSF isolation. CSF samples (1 µl) were diluted 100-fold in PBS, and blood–CSF barrier leakage was determined by measuring the fluorescence (*λ*_ex_/*λ*_em _= 488/520 nm) using the FLUOstar Omega plate reader (BMG Labtech).

### Tissue sample isolation

For RNA analysis, mice were transcardially perfused with a mixture of PBS containing 0.002% heparin sodium (5,000 IU/ml; Wockhardt) and 0.5% bromophenol blue (Sigma-Aldrich; i.e., to macroscopically visualize ChP tissue). Next, ChP (lateral and fourth ventricles), OB, and striatum were dissected from the brain tissue. Isolated samples were snap-frozen in liquid nitrogen for RNA or protein analysis. For immunohistochemical analysis, mice were transcardially perfused with PBS containing 0.002% heparin sodium followed by 1% PFA after which brain samples were postfixed in 1% PFA ON, followed by paraffin embedding. For wholemount ChP immunostaining, mice were transcardially perfused with a mixture of PBS containing 0.002% heparin sodium. After dissection, the ChP (lateral and fourth ventricles) was postfixed in 4% PFA for 1 h at RT.

### qRT-PCR

RNA was isolated from the ChP, OB, and left-brain striatum using the Aurum Total RNA mini kit (732-6820; Bio-Rad). RNA concentration was measured using the Nanodrop 1000 (Thermo Fisher Scientific), and cDNA was prepared using an iScript cDNA synthesis kit (172-5038; Bio-Rad). qRT-PCR was performed with the LightCycler 480 system (Roche) using the SensiFAST SYBR No-ROX kit (BIO-98002; Bioline). Expression levels of the genes of interest were normalized to the expression of four stable reference genes, consisting of *Hprt*, *Rpl*, *Ubc*, and *Gapdh*, as determined by the geNorm Housekeeping Gene Selection Software (Vandesompele et al., 2002). The expression data are displayed as relative expressions normalized to the mean expression of one condition, as clarified in the figure legends. The primer sequences of the forward and reverse primers for the different genes are provided in Table 1.

**Table 1.** Overview of the sequences of the forward and the reverse primers used for qRT-PCR analysis

**Gene**	**Forward**	**Reverse**
*Hprt*	AGTGTTGGATACAGGCCAGAC	CGTGATTCAAATCCCTGAAGT
*Rpl*	CCTGCTGCTCTCAAGGTT	TGGTTGTCACTGCCTCGTACTT
*Ubc*	AGGTCAAACAGGAAGACAGACGTA	TCACACCCAAGAACAAGCACA
*Gapdh*	TGAAGCAGGCATCTGAGGG	CGAAGGTGGAAGAGTGGGAG
*Il1β*	CACCTCACAAGCAGAGCACAAG	GCATTAGAAACAGTCCAGCCCATAC
*Il6*	TAGTCCTTCCTACCCCAATTTCC	TTGGTCCTTAGCCACTCCTTC
*Tnf*	ACCCTGGTATGAGCCCATATAC	ACACCCATTCCCTTCACAGAG
*C3*	CCAGCTCCCCATTAGCTCTG	GCACTTGCCTCTTTAGGAAGTC

### Western blot

ChP, OB, and left-brain striatum tissues were homogenized in Tris-buffered saline (50 mM Tris HCl, pH 7.5, 150 mM NaCl, 1% Triton X-100 in PBS) supplemented with protease inhibitors (Roche). Protein concentration was measured using the Pierce BCA protein assay (Thermo Fisher Scientific). Equal loading amounts per brain tissue (20 µg for ChP and 80 µg for OB and striatum) or CSF samples [5 or 10 µl, supplemented with protease inhibitors (Roche)] were mixed with sample buffer (0.35 M Tris HCl, pH 6.8, 10% SDS, 35% glycerol, 5% β-mercaptoethanol, 0.5% bromophenol blue) and boiled for 10 min at 95°C. Proteins were separated on a 15% SDS-PAGE gel and transferred to a 0.22 µm nitrocellulose membrane via wet blotting. The nitrocellulose membrane was blocked for 1 h at RT using 1:2 diluted Odyssey blocking buffer (927–40,000; Li-Cor) in PBS, followed by ON incubation at 4°C with the primary antibody anti-α-syn (1:2,000; ab610787; BD Biosciences) in 1:4 diluted Odyssey blocking buffer in PBS. After a washing step, the membrane was incubated for 1 h at RT with the secondary antibody goat anti-mouse DyLight 800 in 1:4 diluted Odyssey blocking buffer in PBS supplemented with 0.01% SDS and 0.1% Tween 20. After washing the membrane, bands were visualized using the Odyssey Fc Imager (Li-Cor). To quantify the protein bands, we subsequently incubated the membranes with β-actin (1:1,000; MA5-15739; Invitrogen) for 1 h at RT followed by incubation with the secondary antibody goat anti-mouse DyLight 680 for 1 h at RT. Image Studio Lite software was used to measure the band intensity, and relative protein levels were obtained after normalization to the respective density of the β-actin band. The expression data for the brain tissues are displayed as relative expression normalized to the mean expression of the α-syn Mono 1 h condition.

### Immunohistochemistry

For immunostainings on mouse brain sections, 5 µm sections were prepared from the paraffin-embedded samples. After paraffin removal, heat-induced antigen retrieval was performed in citrate buffer (H-3300; Vector Laboratories), and endogenous peroxidase activity was blocked by incubating the slides with 3% H_2_O_2_ in methanol for 10 min. Next, the sections were washed with PBS and blocked for 30 min with 5% NGS in PBS containing 0.1% Triton X-100. Subsequently, the sections were incubated ON at 4°C with the primary antibody anti-α-syn (1:500; ab610787; BD Biosciences). After a washing step, sections were incubated with the secondary antibody goat anti-mouse biotin (1:500; E0433; DAKO) for 1 h at RT. Next, amplification of the signal was performed using the ABC system (PK-6100; Vector Laboratories) and TSA (SAT700001EA; Perkin Elmer) according to the manufacturer's instructions, and samples were incubated with streptavidin DyLight 633 for 1 h at RT. Finally, the samples were counterstained with Hoechst (1 µg/ml), and the sections were mounted using 2% n-propyl gallate. Imaging was performed using a Zeiss LSM780 confocal microscope.

For colorimetric stainings on mouse brain sections, 5 µm sections were prepared from the paraffin-embedded samples. After paraffin removal, heat-induced antigen retrieval was performed in citrate buffer (H-3300; Vector Laboratories), and endogenous peroxidase activity was blocked by incubating the slides with 5% H_2_O_2_ in methanol for 30 min at RT. Next, the sections were washed with PBS and blocked for 30 min with 5% normal goat serum in PBS containing 0.5% of BSA and 0.02% Triton X-100, preceded by blocking for 15 min with 1 µg/ml proteinase K in case of the pS129 α-syn staining. Subsequently, the sections were incubated ON at 4°C with the primary antibodies tyrosine hydroxylase (TH; 1:1,000; AB152; Merck Millipore) or pS129 α-syn (1:100; ab51253; Abcam). After a washing step, sections were incubated with the secondary antibody goat anti-rabbit biotin (1:500; E0432; DAKO) for 1 h at RT, and amplification of the signal was performed using the ABC system (PK-6100; Vector Laboratories) for 30 min. After a final wash step with PBS, 3,3-diaminobenzidine (PK-6100; Vector Laboratories) was added for 4 min. Finally, the slides were dehydrated and mounted with a xylene-based mounting medium. Imaging was performed using Zeiss Axio Scan Z1. For the TH staining, automated quantification was performed using QuPath software, and quantification of the percentage brown color was done via color thresholding with correction for the total tissue area.

For immunostainings on wholemount ChP, the ChP tissue was postfixed for 1 h in 4% PFA at RT immediately after isolation followed by washing with PBS and blocking with 1% BSA for 1.5 h at RT. Next, samples were incubated ON at 4°C with the primary antibody anti-α-syn (1:500; ab610787; BD Biosciences). After washing, the tissue was incubated ON with the secondary antibody goat anti-mouse DyLight 488 at 4°C. Next, washing followed by a counterstaining with Hoechst (1 µg/ml) for 20 min at RT was performed. Following the final wash steps, the ChP tissue was mounted on iBidi 8-well chambers coated with Cell-Tak (3.5 µg/cm^2^ surface area in sodium bicarbonate buffer) and covered with glycerol mounting medium. Imaging was performed using a Zeiss LSM780 confocal microscope.

### Motor function tests

Motor function was assessed using the challenging beam traversal, pole descent, and inverted grid test as previously described (Fleming et al., 2004; Sampson et al., 2016; Berlamont et al., 2021). The mice were continuously habituated in the testing room to be familiarized with the surroundings. To exclude the interference of olfactory clues, we thoroughly cleaned all objects with 20% ethanol after each trial.

For the beam traversal test, a challenging beam with a total length of 1 m, composed of four segments with consecutively thinner widths (i.e., 3.5, 2.5, 1.5, and 0.5 cm), and containing 1 cm overhangs (placed 1 cm below the surface of the beam) at its beginning and end was placed on top of three inverted mouse cages. The home cage of the mice performing the test was placed on its side so that the narrowest end of the beam led right into the cage. The complete procedure consisted of 2 training days and 1 testing day, separated by 24 h intervals. During the first training day, each mouse had to traverse the beam five times. In the first assisted trial, the home cage containing cage mates was used to guide the mouse along the beam. During the four consecutive trials, mice were gently oriented in the right direction by carefully touching their backside if needed. On the next training day, each mouse again received five trials, whereby they needed minimal assistance to traverse the beam. The following day, the test phase was initiated. Here, a mesh grid (1 cm^2^) with corresponding width to each beam segment was placed on top of the beam whereafter the mice had to traverse the beam five times on top of the grid surface. Each day, all trials were performed per cage of five mice, which resulted in an intertrial interval of approximately 60 s. All trials during the test phase were videotaped and scored manually. Crossing the beam was defined as accomplished when the two front paws entered the home cage. The mean of the three best trials was used to compare the performance of the mice.

For the pole descent test, a 0.5 m long pole with a diameter of 1 cm and wrapped with a nonadhesive shelf liner to facilitate the animals’ grip was placed into the home cage. Again, the complete procedure consisted of 2 training days and 1 testing day. On Day 1, the mice received three trials, whereby they were placed head-down on increasing heights on the pole, starting with 1/3 above the home cage floor followed by 2/3 and ultimately 3/3 or on top of the pole. On both the second training day and the testing day, the mice were given five trials to descend, head-down, from the top of the pole. All trials during the test phase were videotaped and scored manually. Descending the beam was defined as accomplished when both hind limbs touched the home cage floor. The mean of all five trials was used to compare the performance of the mice.

For the inverted grid test, the mice were placed in the center of a 30 cm by 30 cm screen with a 1 cm × 1 cm wide mesh. This screen was inverted and placed on supports approximately 10 cm above an open, acrylic box (40 cm × 40 cm × 40 cm) filled with bedding. The mice were timed manually until they lost their grip or remained on for >120 s.

### Statistics

Statistics was performed using GraphPad Prism 8.3 (GraphPad Software). Data are presented as mean ± SEM. All data were analyzed with a one-way ANOVA unless mentioned differently. Significance levels are indicated on the graphs: *0.01 ≤ *p* < 0.05; **0.001 ≤ *p* < 0.01; ***0.0001 ≤ *p* < 0.001; and ****p < 0.0001.

## Results

### Icv injected human α-syn PFF are removed from the CSF and interact with the ChP, OB, and striatum

Human recombinant α-syn PFF were prepared as described previously (Volpicelli-Daley et al., 2014), followed by quality control according to the current recommendations in the field (Polinski et al., 2018). After incubation for 7 d at 37°C, the PFF solution appeared turbid, presenting as long fibrils with a mean size of 107.95 nm on TEM (Extended Data Fig. 1-1*E*,*G*) and generating a higher signal in the ThT assay compared with Mono (Extended Data Fig. 1-1*A*). Following ultracentrifugation, analysis via denaturing SDS-PAGE revealed more α-syn protein in the pellet versus the supernatant for the PFF, whereas the reverse pattern was observed for the Mono (Extended Data Fig. 1-1*B*). In line with the latter result, TEM on Mono did not reveal the presence of aggregated protein (Extended Data Fig. 1-1*D*). After sonication, TEM showed the presence of shorter PFF with an average length of 44.64 nm (Extended Data Fig. 1-1*F*,*H*). The seeding capacity of the sonicated PFF was confirmed in a ThT assay showing the recruitment of Mono by PFF species, whereby sonicated PFF seed fibril growth more efficiently than unsonicated PFF (Extended Data Fig. 1-1*C*). Additionally, sonicated PFF but not Mono were able to convert endogenous α-syn into aggregated α-syn phosphorylated at serine 129 (pS129 α-syn) in primary hippocampal neurons (Extended Data Fig. 1-1*I*), again confirming their seeding capacity.

**Figure 1.**
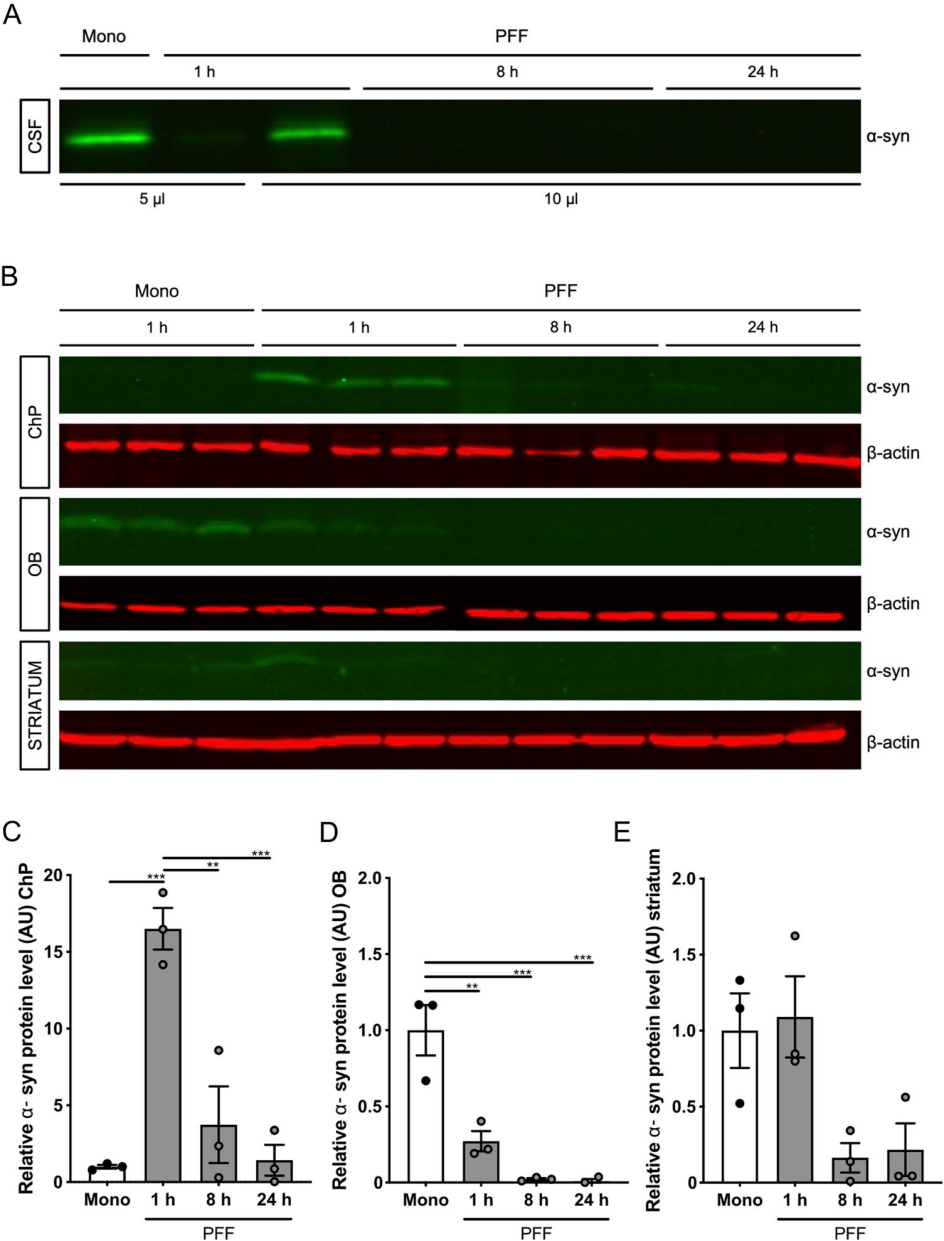
Analysis of the spreading of human α-synuclein (α-syn) preformed fibrils (PFF) and monomers (Mono) after their intracerebroventricular (icv) injection. ***A***, WB analysis for α-syn in the CSF 1 h after the icv injection of Mono and 1, 8, and 24 h after the icv injection of PFF. The loading volumes are mentioned underneath the blot. ***B***, WB analysis for α-syn in the ChP (20 µg), OB (80 µg), and striatum (80 µg) 1 h after the icv injection of Mono and 1, 8, and 24 h after the icv injection of PFF. **C**–**E**, Quantification of α-syn protein levels in the ChP (***C***), OB (***D***), and striatum (***E***) 1 h after the icv injection of Mono (black) and 1, 8, and 24 h after the icv injection of PFF (gray). Protein levels in AU were normalized to β-actin levels and represented relative to the Mono 1 h condition (*n* = 3). See Extended Data Figure 1-1 for characterization of human a-synuclein (α-syn) PFF.

Next, C57BL/6J mice were injected into their left lateral brain ventricle with either sonicated PFF (from here on referred to as PFF) or Mono, the latter being the most recommended control representing the start material from which the PFF are generated (Polinski et al., 2018). At several timepoints after injection (i.e., 1 h for Mono and 1, 8, and 24 h for PFF), CSF was collected and analyzed for the presence of α-syn via Western blot (WB). As presented in Figure 1*A*, more Mono than PFF remained in the CSF 1 h after icv injection. At 8 and 24 h after injection, PFF could no longer be detected in the CSF. To investigate whether the Mono and PFF are taken up by the brain, we analyzed the presence of α-syn in several brain regions, namely, the ChP, OB, and striatum. This revealed the highest α-syn signal at the ChP 1 h after PFF injection, while Mono could not be detected at this timepoint (Fig. 1*B*,*C*). In contrast, the reverse was observed in the OB where more Mono than PFF were seen 1 h after injection (Fig. 1*B*,*D*). In the striatum, an equal signal for Mono and PFF was present at this timepoint (Fig. 1*B*,*E*). At later timepoints after PFF injection, the signal steeply decreased in the ChP, whereas barely any signal was detectable in both the OB and striatum.

10.1523/ENEURO.0024-23.2024.f1-1 Figure 1-1 **Characterization of human alpha synuclein (α-syn) pre-formed fibrils (PFF).** Thioflavin T (ThT) assay of 0.05 mg/ml monomers (Mono) or PFF to confirm the presence of typical β-sheet structures in PFF but not in Mono. **(B)** Denaturing sodium dodecyl sulfate (SDS) polyacrylamide gel electrophoresis and Coomassie stain for volumetric analysis of the supernatant fraction (SN) and pellet fraction (P) of PFF and Mono, post-centrifugation. **(C)** ThT kinetic seeding assay to investigate recruitment of Mono by PFF. Seeds (unsonicated or sonicated PFF) (1 mg/ml) were added at a ratio of 1:20 to Mono (0.05 mg/ml). **(D-F)** Representative transmission electron microscopy (TEM) images of Mono (D), unsonicated PFF (E) and sonicated PFF (F). Scale bar represents 100 nm (D) or 200 nm (E, F). **(G-H)** Quantification of unsonicated (G) and sonicated (H) PFF fibril length measured from the representative TEM images. **(I)** Representative confocal images of pS129 α-syn (red) in primary hippocampal neurons after 11 days of incubation with Mono or PFF (n=3). Cell nuclei are counterstained with Hoechst (blue) and neurons are counterstained with β-III-tubulin (green). Scale bar represents 20 µm. Download Figure 1-1, TIF file.

**Figure 2.**
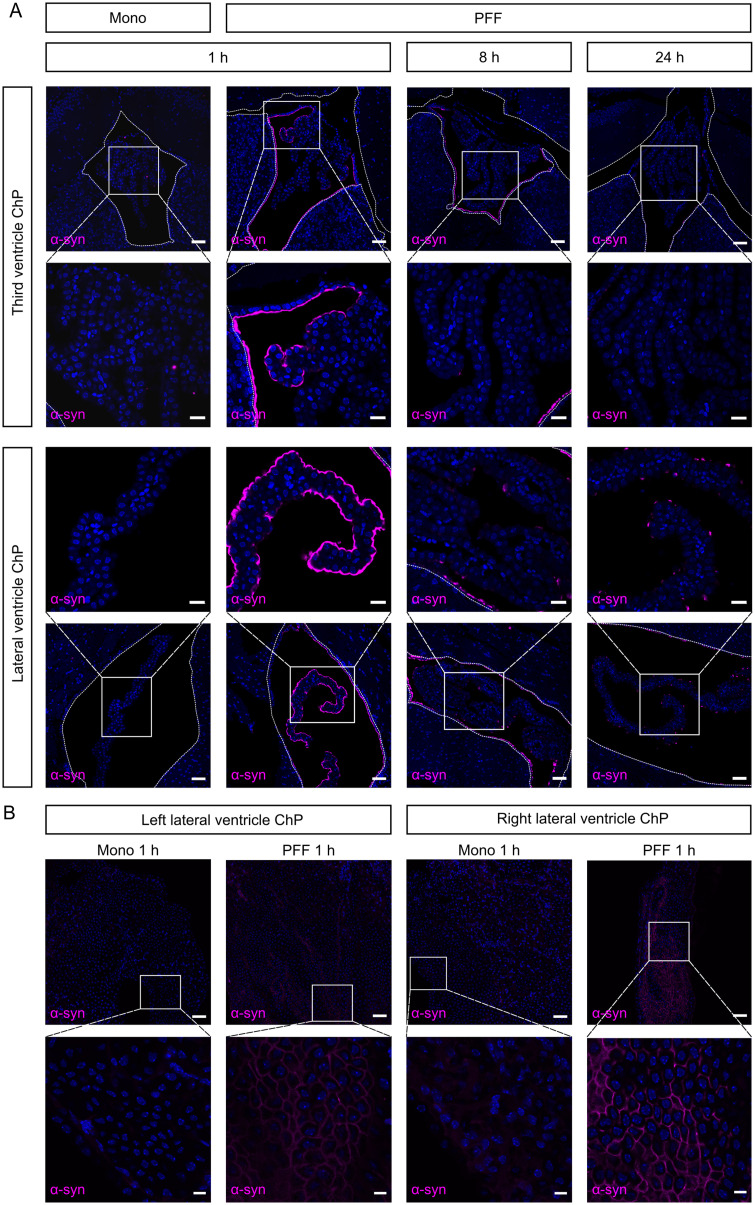
Analysis of the intraventricular localization of α-synuclein (α-syn) after the intracerebroventricular (icv) injection of human α-syn preformed fibrils (PFF) and monomers (Mono). ***A***, Representative confocal images of α-syn (magenta) in the choroid plexus (ChP) of the third and the lateral ventricle 1 h after the icv injection of Mono and 1, 8, and 24 h after the icv injection of PFF (*n* = 3). Cell nuclei are counterstained with Hoechst (blue). The ependymal cells that line the ventricle are indicated by a white line. The scale bar represents 50 µm on the overview images and 20 µm on the zoomed-in images. See Extended Data Figure 2-1 for a *z*-stack reconstruction of α-syn in the ChP of the lateral ventricle 1 h after the icv injection of PFF. ***B***, Representative confocal images of α-syn (magenta) in ChP wholemounts of the left and right lateral ventricle 1 h after the icv injection of Mono or PFF (*n* = 2). Cell nuclei are counterstained with Hoechst (blue). The scale bar represents 50 µm on the overview images and 10 µm on the zoomed-in images.

To further investigate the interaction between α-syn and the ChP, we performed immunofluorescent staining on brain sections derived from mice icv injected with either Mono or PFF (Fig. 2*A*). This revealed the absence of Mono in the ChP 1 h after their icv injection, confirming the WB results. In contrast, the PFF could clearly be detected in association with the ChP and the ependymal cell layer lining the ventricles 1 h after injection. A *z*-stack reconstruction indicated that the PFF stick to the surface of the ChP epithelial and ependymal cells rather than building up intracellularly as shown by the absence of cytoplasmic signal (Extended Data Fig. 2-1). Eight hours after injection, PFF could still be detected at the ependymal cells, whereas the signal in the ChP was almost completely absent except for some dotted signal on top of certain cells, mainly in the lateral ventricle ChP (Fig. 2*A*). This was also still present 24 h after injection, whereas the ependymal signal declined further toward being almost absent. No signal could be detected in the OB or in the striatum in any of the conditions. Similar to the α-syn visualization on paraffin sections, also wholemount stainings on ChP tissue isolated from Mono- and PFF-injected mice 1 h after the icv injection revealed that almost no signal was present in the ChP tissue isolated from Mono-injected mice. In contrast, ChP tissue from PFF-injected mice showed a clear α-syn signal representing a honeycomb-like pattern between and on top of the ChP epithelial cells (Fig. 2*B*).

10.1523/ENEURO.0024-23.2024.f2-1 Figure 2-1 **Analysis of the intraventricular localization of alpha-synuclein (α-syn) 1 h after the intracerebroventricular (icv) injection of human alpha synuclein (α-syn) pre-formed fibrils (PFF).** Z-stack reconstruction of α-syn (magenta) in the choroid plexus (ChP) of the lateral ventricle 1 h after the icv injection of PFF (n=3). Cell nuclei are counterstained with Hoechst (blue). Scale bar (x-, y-, z-axis) represents 20 µm per square. Download Figure 2-1, TIF file.

### Icv injected human α-syn PFF induce an inflammatory response in the ChP, OB, and striatum

**Figure 3.**
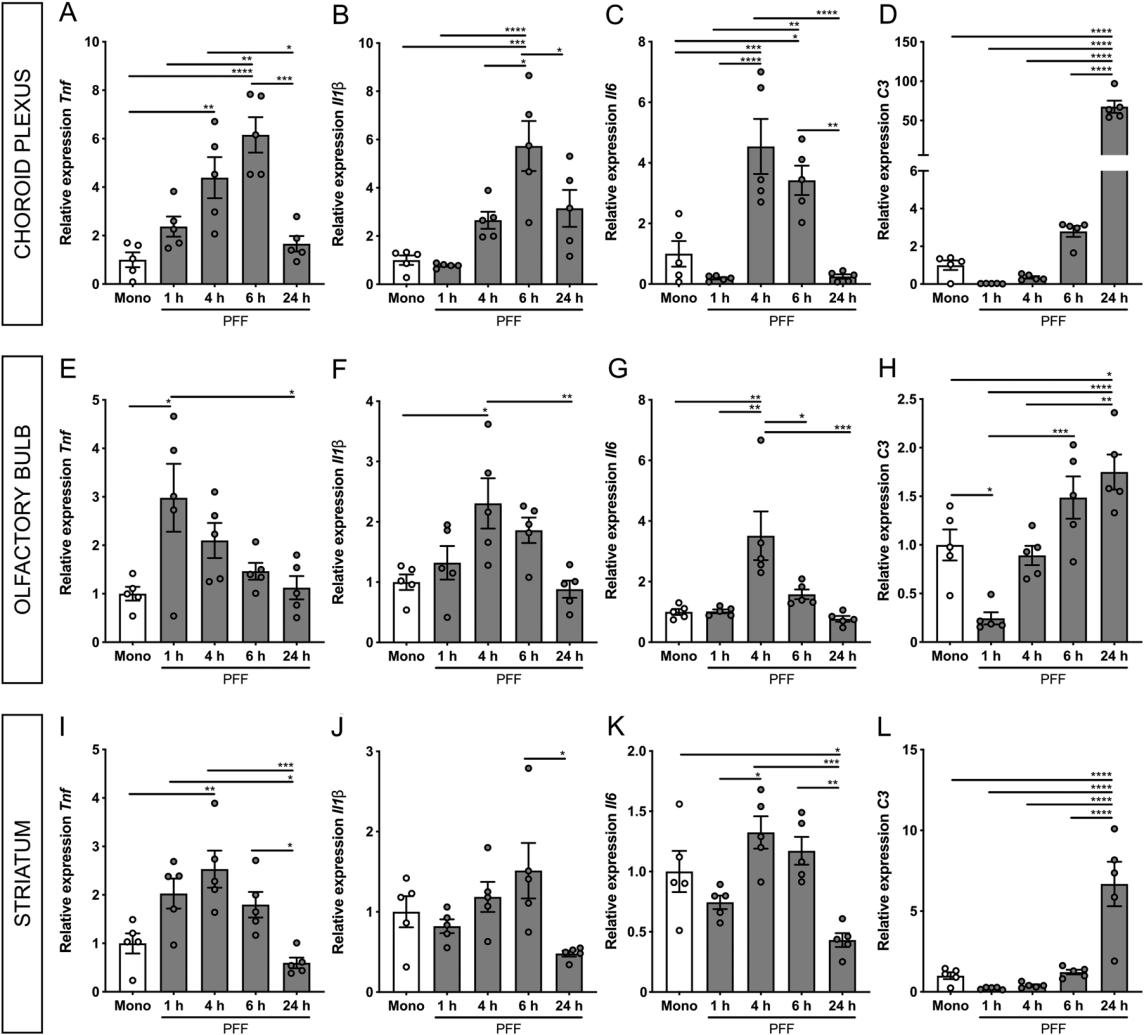
Analysis of inflammatory and complement gene expression in the choroid plexus (ChP), olfactory bulb (OB), and striatum after the intracerebroventricular (icv) injection of human α-synuclein (α-syn) preformed fibrils (PFF) and monomers (Mono). ***A***–***D***, qRT-PCR analysis of *Tnf* (***A***), *Il1β* (***B***), *Il6* (***C***), and *C3* (***D***) in the ChP 6 h after the icv injection of Mono (black) and 1, 4, 6, and 24 h after the icv injection of PFF (gray), represented relative to the Mono 6 h condition (*n* = 5). ***E***–***H***, qRT-PCR analysis of *Tnf* (*E*), *Il1β* (F), *Il6* (*G*), and *C3* (*H*) in the OB 6 h after the icv injection of Mono (black) and 1, 4, 6, and 24 h after the icv injection of PFF (gray), represented relative to the Mono 6 h condition (*n* = 5). ***I***–***L***, qRT-PCR analysis of *Tnf* (***I***), *Il1β* (***J***), *Il6* (***K***), and *C3* (***L***) in the OB 6 h after the icv injection of Mono (black) and 1, 4, 6, and 24 h after the icv injection of PFF (gray), represented relative to the Mono 6 h condition (*n* = 5). See Extended Data Figures 3-1 and 3-2 for comparisons of the effects induced by human α-syn PFF, Mono, and PBS 1 h and 6 h after their icv injection.

Next, we examined the inflammatory response in the ChP, OB, and striatum in response to PFF in the CSF. To this end, we analyzed the gene expression levels of *Tnf*, *Il1β*, and *Il6* using qRT-PCR analysis. In these experiments, also the Mono 6 h timepoint was included because preliminary data indicated that Mono had an impact on the ChP independent of the icv injection procedure (Extended Data Fig. 3-1). In the ChP, the levels of all analyzed inflammatory cytokines were significantly upregulated with a peak expression of either 4 h (*Il6*) or 6 h (*Tnf* and *Il1β*) after PFF injection in comparison with both the Mono and PFF 1 h groups. The levels were normalized again after 24 h (Fig. 3*A*–*C*). In the OB, the peak expression of *Tnf* and *Il1β* occurred earlier (after 1 and 4 h, respectively) in comparison with the ChP, whereas the pattern for *Il6* is similar (Fig. 3*E*–*G*). Similarly, the expression pattern in the striatum (Fig. 3*I*–*K*) was comparable with the pattern observed in the ChP. However, the most pronounced proinflammatory effect induced by the PFF was observed in the ChP (Fig. 3*A*–*C*) and less in the OB (Fig. 3*E*–*G*) and in the striatum (Fig. 3*I*–*K*).

10.1523/ENEURO.0024-23.2024.f3-1 Figure 3-1 **Comparison of the effects induced by human alpha synuclein (α-syn) pre-formed fibrils (PFF), monomers (Mono) and PBS 1h and 6 h after their intracerebroventricular (icv) injection. (A-D)** qRT-PCR analysis of *Tnf* (A), *Il1β* (B), *Il6* (C) and *C3* (D) in the choroid plexus (ChP) 1 h and 6 h after the icv injection of PBS (light grey), Mono (black) or PFF (grey). Data are represented relative to the 1 h timepoint for each treatment condition (n=5). **(E)** Blood-CSF barrier permeability 1 h and 6 h after the icv injection of PBS (light grey), Mono (black) or PFF (grey) determined by intravenous (iv) injection of fluorescently labelled 4 kDa dextran followed by analysis of the fluorescence in the CSF. Data are represented relative to the 1 h timepoint for each treatment condition (n=5). Download Figure 3-1, TIF file.

Next to proinflammatory cytokines, we also looked at the expression of the complement 3 (*C3*) gene since emerging evidence suggests that complement activation plays a role in PD (Carpanini et al., 2019). As shown in Figure 3*D*, the *C3* expression is extensively upregulated in the ChP 24 h after the icv injection of PFF. However, to a lesser extent, this is also the case in the OB (Fig. 3*H*) and striatum (Fig. 3*L*).

Next to Mono as a control, we also included a group of mice injected with PBS (i.e., α-syn vehicle) to be able to compare the Mono effect to the effect of the icv injection procedure itself. The comparison between gene expression in the tissues 6 h after the injection of either PBS, Mono, or PFF is represented in Extended Data Figure 3-2. Although the Mono induce a higher inflammatory gene expression in comparison with the PBS group, the PFF effect was, in general, the most pronounced (Extended Data Fig. 3-2*A*–*L*), indicating that also the conformation of the α-syn species plays a role in provoking a proinflammatory response.

10.1523/ENEURO.0024-23.2024.f3-2 Figure 3-2 **Comparison of the effects induced by human alpha synuclein (α-syn) pre-formed fibrils (PFF), monomers (Mono) and PBS 6 h after their intracerebroventricular (icv) injection. (A-D)** qRT-PCR analysis of *Tnf* (A), *Il1β* (B), *Il6* (C) and *C3* (D) in the choroid plexus (ChP) 6 h after the icv injection of PBS (light grey), Mono (black) or PFF (grey). Data are represented relative to the PBS condition (n=5). **(E-H)** qRT-PCR analysis of *Tnf* (E), *Il1β* (F), *Il6* (G) and *C3* (H) in the olfactory bulb (OB) 6 h after the icv injection of PBS (light grey), Mono (black) or PFF (grey). Data are represented relative to the PBS condition (n=5). **(I-L)** qRT-PCR analysis of *Tnf* (I), *Il1β* (J), *Il6* (K) and *C3* (L) in the striatum 6 h after the icv injection of PBS (light grey), Mono (black) or PFF (grey). Data are represented relative to the PBS condition (n=5). **(M)** Blood-CSF barrier permeability 6 h after the icv injection of PBS (light grey), Mono (black) or PFF (grey) determined by intravenous (iv) injection of fluorescently labelled 4 kDa dextran followed by analysis of the fluorescence in the CSF. Data are represented relative to the PBS condition (n=5). Download Figure 3-2, TIF file.

### Icv injected human α-syn PFF induce leakage of the blood–CSF barrier

**Figure 4.**
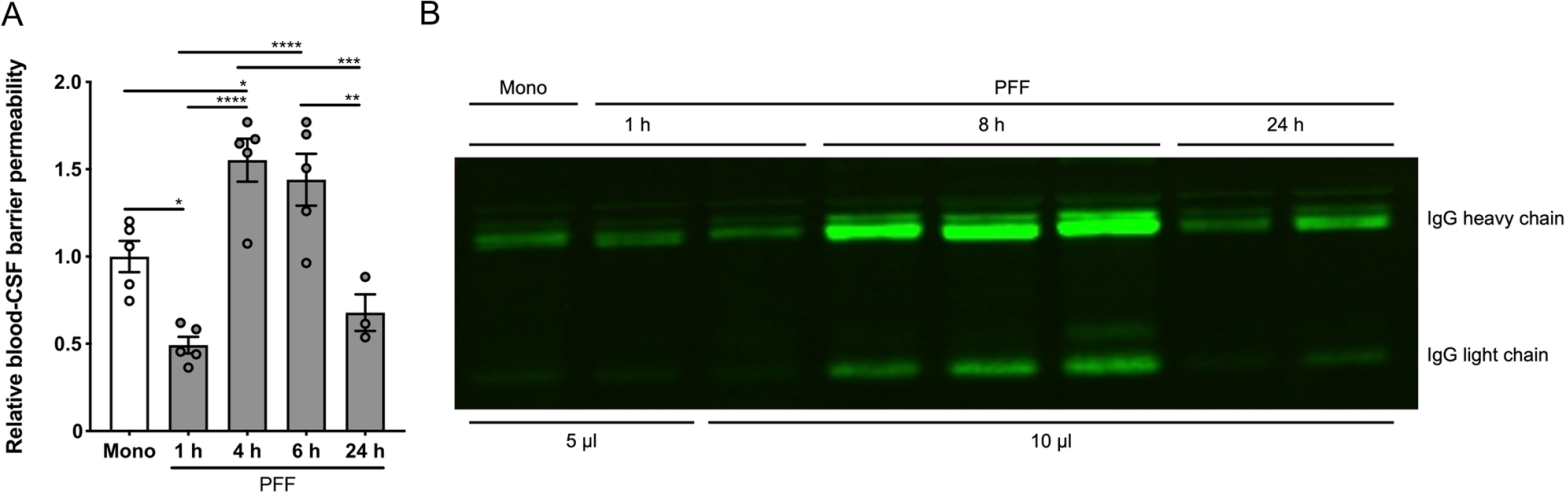
Analysis of the blood–cerebrospinal fluid (CSF) barrier leakage after the intracerebroventricular (icv) injection of human α-synuclein (α-syn) preformed fibrils (PFF) and monomers (Mono). ***A***, Blood–CSF barrier permeability 6 h after the icv injection of Mono (white) and 1, 4, 6, and 24 h after the icv injection of PFF (gray) determined by intravenous (iv) injection of fluorescently labeled 4 kDa dextran followed by an analysis of the fluorescence in the CSF. Data are represented relative to the Mono 6 h condition (*n* = 3–5). ***B***, WB analysis for IgG in the CSF 1 h after the icv injection of Mono and 1, 8, and 24 h after the icv injection of PFF. The loading volumes are mentioned underneath the blot.

Previously, it has been shown that icv injection of Aβ oligomers not only induces an inflammatory response in the ChP but also causes loss of blood–CSF barrier integrity (Brkic et al., 2015; Steeland et al., 2018). To investigate this, we injected 4 kDa FITC-dextran intravenously 15 min before CSF isolation of mice that were icv injected with Mono or PFF at different timepoints prior to sampling. This revealed significantly increased blood–CSF barrier permeability 4 and 6 h after the icv injection of PFF in comparison with the 1 and 24 h timepoints (Fig. 4*A*), indicating that PFF induce a transient leakage of the blood–CSF barrier. These data were confirmed via WB analysis of CSF samples, which revealed a higher IgG signal 8 h after icv injection of PFF compared with the 1 and 24 h timepoints (Fig. 4*B*). Although Mono also induced some loss of barrier integrity compared with PBS (Extended Data Fig. 3-2*M*), the impact of PFF is significantly more pronounced.

### Icv injected human α-syn PFF induce a PD-like phenotype

Next, we studied whether the icv injection of PFF could induce a cascade of α-syn aggregation and spreading associated with the occurrence of motor symptoms. Hereby, we performed several well-characterized and broadly used motor function tests on the icv-injected mice for several months. Importantly, the control group was icv injected with PBS as it has been described that Mono induce mild pathology in the long term (Paumier et al., 2015). As shown in Extended Data Figure 5-1, a single injection of PFF failed to induce a phenotype as evidenced by the absence of motor function deficits assessed by time to cross the beam in the beam traversal test (Extended Data Fig. 5-1*A*) and time to descend the pole in the pole descent test (Extended Data Fig. 5-1*B*). Furthermore, no deterioration in grip strength assessed by the time to fall in the inverted grid test (Extended Data Fig. 5-1*C*) was present, even 8 months after the icv injection of PFF.

**Figure 5.**
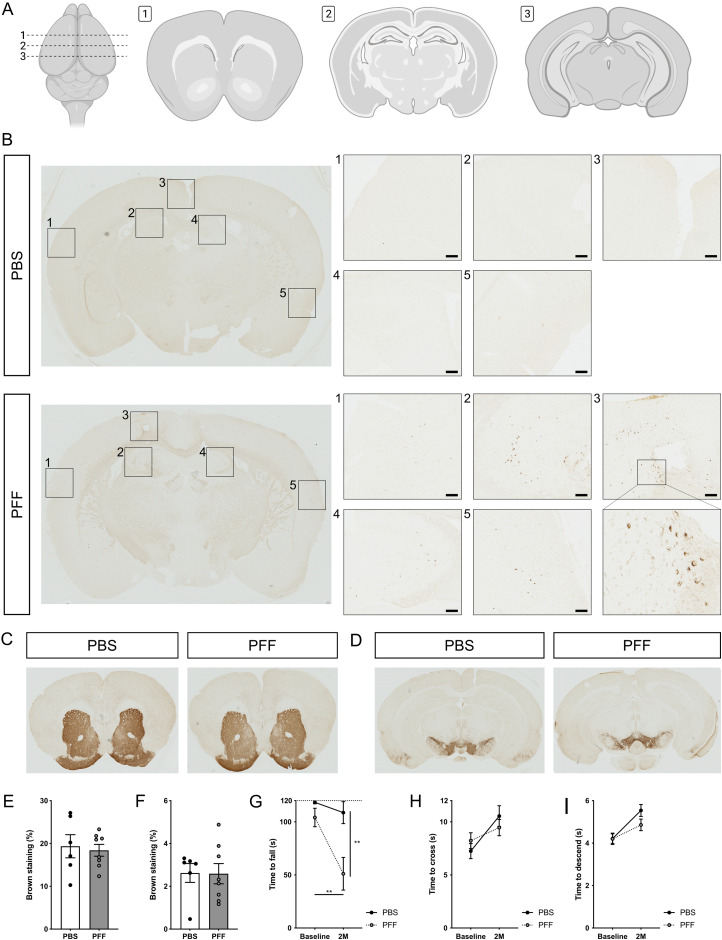
Analysis of a Parkinson's disease (PD)-like phenotype 2 months after the repeated intracerebroventricular (icv) injection (five injections over 5 d) of human α-synuclein (α-syn) preformed fibrils (PFF) and PBS. See Extended Data Figure 5-2 for an overview of the experimental setup of this experiment. ***A***, Overview of brain sectioning after sampling. Section 1 contains the striatum, Section 2 contains the icv injection site, and Section 3 contains the substantia nigra. ***B***, Representative images of α-syn phosphorylated at serine 129 (pS129 α-syn; brown) in the brain (Section 2) 2 months after the repeated icv injection of PBS (*n* = 6) or PFF (*n* = 8). The scale bar represents 100 µm. ***C***, ***D***, Representative images of TH staining for dopaminergic neuron fiber density in the striatum (***C***, Section 1) and the substantia nigra (***D***, Section 3) 2 months after the repeated icv injection of PBS (*n* = 6) or PFF (*n* = 8). ***E***, ***F***, Quantification of dopaminergic neuron fiber density based on TH staining in the striatum (***E***) and the substantia nigra (***F***) 2 months after the repeated icv injection of PBS (black, *n* = 6) or PFF (gray, *n* = 8). ***G***–***I***, Inverted grid (***G***), beam traversal (***H***), and pole descent (***I***) at baseline and 2 months after the repeated icv injection of PBS (black, *n* = 6) or PFF (gray, *n* = 8). See Extended Data Figure 5-1 for the analysis of motor function after a single icv injection of human α-syn PFF and PBS.

Next, to maintain a higher PFF concentration over a prolonged time course, we icv injected mice for 5 consecutive days with either PBS or PFF (Extended Data Fig. 5-2). In this setup, we observed deposits of pS129 α-syn around the injection site, in the hippocampal area, and in the cortex 2 months after the injection of PFF, but not PBS (Fig. 5*B*). In contrast, no loss of TH immunoreactivity was observed in the striatum (Fig. 5*C*,*E*) nor in the substantia nigra (Fig. 5*D*,*F*). Nonetheless, PFF-injected mice showed a significantly decreased time to fall in the inverted grid test, both in comparison with baseline testing and with PBS-injected mice 2 months after injection (Fig. 5*G*). This indicates that PFF-injected mice experience a reduced grip strength or a reduced fine motor skill causing them to fall when moving around the grid. In contrast, no differences were observed in the beam traversal test (Fig. 5*H*) nor in the pole descent test (Fig. 5*I*).

10.1523/ENEURO.0024-23.2024.f5-1 Figure 5-1 **Analysis of motor function after the intracerebroventricular (icv) injection of human alpha synuclein (α-syn) pre-formed fibrils (PFF) and PBS. (A-C)** Beam traversal (A), pole descent (B) and inverted grid (C) test at several timepoints after the icv injection of human PFF (grey) or PBS (black) (n=8). Download Figure 5-1, TIF file.

10.1523/ENEURO.0024-23.2024.f5-2 Figure 5-2 **Overview of experimental setup of repeated intracerebroventricular (icv) injection experiment.** At baseline (day 0), mice were first assessed using the beam traversal test, followed by the pole descent test and finally the inverted grid test. Training for the beam traversal test and pole descent test was performed on the days preceding day 0 following the schedule as described in the materials and methods section. From day 1 until day 5, mice were icv injected daily with either 5 µl of 1 μg/μl human alpha synuclein (α-syn) pre-formed fibrils (PFF) or an equal volume of PBS. Approximately 2 months later, on day 77, mice were again assessed using the beam traversal test, followed by the pole descent test and finally the inverted grid test. Training for the beam traversal and pole descent test was not repeated at this timepoint. On day 80, mice were sacrificed. Download Figure 5-2, TIF file.

## Discussion

One of the hallmarks of PD is the misfolding and aggregation of the protein α-syn, which is the major component of the pathognomonic Lewy bodies located intracellularly in neurons. Additionally, α-syn is present in human biofluids including CSF, whereby neurons are believed to be the principal source (Mollenhauer et al., 2012). PD patients harbor decreased total α-syn but increased oligomeric α-syn levels in their CSF in comparison with control groups (Zhou et al., 2015; Eusebi et al., 2017; Parnetti et al., 2019). Additionally, protein amplification assays indicate the presence of misfolded α-syn seeds with self-aggregating properties in CSF samples from synucleinopathy patients (Fairfoul et al., 2016; Shahnawaz et al., 2016; Groveman et al., 2018; Rumund et al., 2019; Rossi et al., 2020) and even in prodromal PD patients (Siderowf et al., 2023), which may underline the importance of CSF α-syn seeds even early on in PD pathology. Furthermore, the FluoReSyn tool (i.e., a fluorescent reporter for human α-syn) indicates that α-syn present in human CSF is able to enter into FluoReSyn reporter cells, pointing toward a potential role of the CSF in mediating α-syn transmission (Gerdes et al., 2020). Together, these results clearly support the presence of α-syn aggregates harboring seeding capacity in CSF of PD patients. Nevertheless, not much is known about the distribution of pathogenic α-syn species throughout the CSF and their effect on surrounding brain structures, even though these findings could lead to novel strategies to impact α-syn spreading through the brain or attenuate brain deterioration in the future. To investigate this, we combined the well-established seeding-competent human recombinant α-syn PFF (Polinski et al., 2018) with an icv injection mouse model. Hereby, we injected α-syn PFF or Mono into the CSF of the left lateral ventricle whereafter we anticipated their spreading as described in the literature, that is, from the lateral ventricles to the third ventricle, followed by the fourth ventricle and ultimately via the cisterna magna in the CSF surrounding the brain. One hour after the icv injection, WB analysis indicated that more Mono than PFF remained in the CSF, which might be indicative of the presence of a specific uptake mechanism for PFF. To this end, the ChP that is hanging inside the ventricle provides an interesting candidate due to its transporter function and a unique location between the CSF and the blood (Kratzer et al., 2020). For instance, in the context of AD, it was shown that ChP explants derived from rat brains are able to take up Aβ_1-40_ from artificial CSF (Crossgrove et al., 2005). Additionally, we and others have shown that ChP epithelial cells can transfer Aβ_1-40/42_ from the inner (i.e., CSF side) to the outer compartment (i.e., blood side) of a transwell system (Gu et al., 2014; Xie et al., 2021). Of particular interest, using the latter setup similar results were obtained with α-syn as well (Bates et al., 2015). Moreover, postmortem sections of patients with synucleinopathies including PD patients exhibit α-syn reactivity within their ChP (Mollenhauer et al., 2012). Together, these clues led us to investigate whether the ChP is involved in the uptake of α-syn from the CSF in our model. Strikingly, 1 h after the icv injection, we could detect a clear association of PFF with the ChP as evidenced by WB analysis on ChP tissue and immunofluorescent stainings on both brain sections and ChP wholemount tissue. The latter two analyses indicated that α-syn closely interacts with the ChP epithelial plasma membrane, while uptake is rather limited. Thereby, this result is more indicative of an interaction mechanism of the PFF with the ChP rather than an uptake mechanism of the PFF by the ChP. Next to the ChP, the α-syn signal could also be detected along the ependymal cell layer lining the ventricles. Importantly, icv injection of the same concentration of Mono showed a completely different picture: α-syn could not be observed in the ChP nor with the ependymal cell layer. It is therefore tempting to speculate that the ChP might be able to specifically interact with pathogenic α-syn species, which might impact their CSF clearance from the brain.

In addition to the ChP, we investigated the distribution of the injected α-syn to two other highly interesting brain structures in the context of PD, namely, the OB and striatum. The former represents one of the first affected brain regions in the disease process (Braak et al., 2003), whereas the latter is the brain region responsible for generating the motor output of the dopaminergic neurons that project from the substantia nigra (Gantz et al., 2018). The OB is closely linked with one of the drainage routes of the CSF from the brain. Indeed, from the fourth ventricle, the CSF enters the SAS from where it is either reabsorbed into the bloodstream or into the peripheral lymphatic system (Spector et al., 2015; Simon and Iliff, 2016). The latter can occur via the cribriform plate and nasal lymphatics, as well as via lymphatic vessels in the dura mater and along cranial and spinal nerve roots (Engelhardt et al., 2017). Interestingly, fluorescent tracers infused into the cisterna magna of mice are known to enter the SAS whereafter they leave the brain via the cribriform plate and the nose (Bedussi et al., 2015). Additionally, a small molecular weight tracer of 3 kDa could enter the brain parenchyma from the SAS by crossing the pia mater, which was particularly evident around the OB (Bedussi et al., 2015). In our setup, we observed the presence of a significantly higher amount of Mono in comparison with PFF in the OB 1 h after the icv injection. This flow direction is in line with the fluorescent tracer study, whereby the higher amount of Mono that reaches the OB in our model might be explained by the higher amount of Mono that remain in the CSF due to their lack of interaction with the ChP. The CSF drainage route via the OB might be of particular importance for the pathogenesis of PD since the OB often represents the first region where α-syn aggregates appear according to the Braak staging (Braak et al., 2003). In the striatum, located immediately adjacent to the lateral ventricles, only a very weak α-syn signal was present in both the PFF and Mono conditions. This might be the result of the rapid flow of the α-syn species from their injection site (i.e., the left lateral ventricle) toward the third and fourth ventricles. Comparing the α-syn signal between different tissues may however be confounded by the size of the tissues (i.e., more diluted α-syn signal in larger tissues), indicating that less detectable α-syn signal does not exclude pathological relevance.

Next, to the spreading of the injected α-syn toward several brain regions of interest, we investigated the inflammatory response in these tissues. The involvement of inflammation in the course of PD is underlined by a variety of studies reporting that the levels of several proinflammatory cytokines (e.g., TNF, IL-1β, IL-6) are increased in the brain and in the CSF of PD patients (Mogi et al., 1996; Brodacki et al., 2008; Karpenko et al., 2018; Lin et al., 2019; Tan et al., 2020). Hereby, α-syn itself is proposed as an important trigger of the immune response (Reish and Standaert, 2015). We observed that the icv injection of PFF induced a transient proinflammatory response that was most pronounced in the ChP but also occurred in the OB and striatum. In general, the proinflammatory response induced by PFF was significantly higher in comparison with Mono. However, this was particularly evident in the ChP but less obvious in the OB and striatum. These results are in line with the observed distribution pattern of the injected α-syn species, that is, significantly more PFF than Mono in the ChP (represented by a more pronounced PFF effect on inflammatory gene expression), significantly less PFF than Mono in the OB, and an equal amount of PFF and Mono in the striatum (both represented by a more equal Mono and PFF effect on inflammatory gene expression). This suggests that the elicited proinflammatory response is induced by α-syn itself and that PFF harbor a higher proinflammatory capacity in comparison with Mono. Of note, also Mono induced a higher inflammatory gene expression in comparison with mice injected with PBS, a control group for the icv injection procedure itself, although the PFF effect was in general the most pronounced. Nonetheless, we can't pinpoint the specificity of this response to the icv injection of PFF since we have previously shown that also the icv injection of AβO affects the functionality of the blood–CSF barrier (Brkic et al., 2015; Steeland et al., 2018; Vandendriessche et al., 2021). However, our data allow us to conclude that not any protein that is introduced into the CSF will impact the ChP to the same extent but that different disease-specific proteins known to be present in the CSF of patients (e.g., AβO in case of AD patients or α-syn in case of PD patients) may cause similar effects.

On top of the inflammatory response in the ChP, we also observed an acute and transient loss of blood–CSF barrier integrity as assessed by 4 kDa FITC-dextran leakage into the CSF, similarly as has been reported upon the icv injection of Aβ oligomers (Brkic et al., 2015; Steeland et al., 2018). Although very little is known about the blood–CSF barrier in PD pathogenesis, two studies investigated the ratio of albumin in lumbar CSF versus serum as a readout for blood–CSF barrier leakage. Hereby, an increased ratio was seen in PD patients with advanced disease in comparison with early-disease PD patients and healthy controls (Haussermann et al., 2001; Pisani et al., 2012). Although not conclusive, these data suggest an increased blood–CSF barrier permeability along the course of PD, which according to our data could be caused by the presence of seeding-competent α-syn in the CSF.

Emerging evidence suggests that complement activation plays a role in PD as well. Indeed, several studies reveal increased staining of various complement proteins on Lewy bodies in the substantia nigra of PD patients and patients with Lewy body dementia in comparison with age-matched controls (Yamada et al., 1992; Iseki et al., 2000; Loeffler et al., 2006). Furthermore, the serum levels of several complement factors are increased in PD patients, whereby increased C3 and C4 levels are linked with worse quality of life and memory ability (Goldknopf et al., 2006; Veselý et al., 2018). Here, we could show that PFF exert a profound effect on *C3* gene expression in the ChP, OB, and striatum, especially at the 24 h timepoint. Strikingly, the fold change of the *C3* upregulation in the ChP 24 h after icv injection is more than a thousand-fold higher in comparison with the peak expression in *Tnf*, *Il-1β*, and *Il-6*. We know from literature (Singhrao et al., 1999; Moore et al., 2016) and our own single-cell sequencing data (unpublished results) that ChP epithelial cells are able to express complement factor genes, suggesting their local synthesis by the ChP epithelial cells. Additionally, the magnitude and timepoint of the effect might be suggestive of the contribution of peripheral complement proteins, keeping in mind that the liver is its main site of synthesis (Orsini et al., 2014). One of the results of complement activation is the recruitment of immune cells (Hajishengallis et al., 2017). Although speculative and in need of thorough research, it could be suggested that PFF activate the complement pathway in the ChP as a result of the tight interaction of PFF with the ChP. This could be followed by immune cell infiltration into the brain, which could exacerbate CNS inflammation. The observed blood–CSF barrier permeability could further contribute to this process.

In the long term, a single icv injection of human PFF was unable to induce deficits in motor function. This could be the result of clearance of PFF from the CSF, thereby precluding seeding of α-syn pathology. This hypothesis is reinforced by our follow-up experiment, where maintaining a higher PFF concentration over a prolonged time course resulted in a deterioration in grip strength and pS129 α-syn deposition in the brain 2 months post injection although dopaminergic neurons were not affected. In patients, chronic exposure to seeding-competent α-syn over the long disease process might eventually lead to similar results. Of note, in our icv injection paradigm, we can't exclude that small amounts of α-syn seeds were deposited in the cortex along the injection needle tract. However, like most PFF studies in literature, we made use of a 10 µl Hamilton syringe combined with a slow injection rate and a waiting time after injection with the needle in place to minimize this risk. Furthermore, the pS129 α-syn distribution pattern we observe does not correlate with the pattern described by the few studies that used cortical PFF injection as a primary location of injection (Luk et al., 2012; Osterberg et al., 2015). Therefore, we believe that the pS129 α-syn pattern we observe 2 months post injection is mainly caused by seeding following uptake of α-syn PFF by brain regions surrounding the ventricular system and SAS. In literature, a single icv injection of α-syn oligomers, but not Mono or fibrils, was shown to induce an acute but transient memory impairment in the novel object recognition test in mice, whereby these results could be linked to neuroinflammation in the hippocampus (Vitola et al., 2018, 2019, 2020). Using a similar injection paradigm, α-syn oligomers induced a PD-like phenotype consisting of olfactory dysfunction 4 d after injection and motor function deficits 45 d after injection (Fortuna et al., 2017). The differences between the latter study and ours include the gender (male vs female) and genetic background (Swiss vs C57BL/6J) of the mice and the type of α-syn species (α-syn oligomers PFF) that were used.

In conclusion, our data show that icv-injected PFF, but not Mono, are captured from the CSF by the ChP, whereas both Mono and PFF spread toward the OB and striatum. Thereby, especially the PFF induce an inflammatory response in these target tissues. Additionally, the PFF injection is associated with an increase in blood–CSF barrier permeability. In contrast to a single icv injection, five consecutive PFF injections induced a PD-like phenotype and brain pathology. Understanding how the blood–CSF barrier is altered in PD pathogenesis might lead to novel strategies to impact α-syn spreading through the brain or attenuate brain deterioration.

## Synthesis

Reviewing Editor: Mikko Airavaara, Helsingin yliopisto - Viikin kampus Decisions are customarily a result of the Reviewing Editor and the peer reviewers coming together and discussing their recommendations until a consensus is reached. When revisions are invited, a fact-based synthesis statement explaining their decision and outlining what is needed to prepare a revision will be listed below. The following reviewer(s) agreed to reveal their identity: NONE.

This study is focused on "The spreading and effects of human recombinant alpha-synuclein pre-formed fibrils in the cerebrospinal fluid of mice". The authors used in vivo methods to evaluate the impact of alpha-synuclein pre-formed fibrils when injected into the CFS of mice. They replicated data from the literature showing that PFF administration in the lateral ventricle of mice results in long-term PD-like pathology. The literature review in the introduction part does not give enough context for the reader to find the relevance of choroid plexus (CP) in PD pathology. It is quite scarcely written and does not clearly state the study objectives and their novelty. Research gaps are not clearly emphasized in the introduction part of the manuscript, so they should be more clearly written. Future applications of the study results are missing. Therefore, it is hard to see the relevance of this study. The authors should highlight the importance and novelty of their study and its applicability in the future and medicine more clearly.

The experiments appear to be well conducted. However, the results are rather descriptive, and real molecular interactions are not investigated. Finally, the manuscript completely lacks depth beyond describing that α-syn PFF infusion by CFS route is an appropriate model to study PD-like pathology. The relevance of the findings is very questionable.

Methods are overall well described, figures are carefully prepared and body text is well written. However study design suffers from some issues regarding proper control groups, which at least have to be toroughly discussed as limitation. Additionally, the authors have to clarify some other technical question before the manuscript could be considered for publication.

Major:

Validation of proper PFF formation is important, the authors have performed many steps to ensure formation of amyloid, templating aSyn fibirls, with proper size range. However this still have to be validated for ability to induce aggregation in cells. In supplementary figure S1I the authors demonstrate ability of prepared PFFs to cause aggregation in SY5Y cells -however observed pS129-aSyn formed rather fuzzy image rather than dense aggregates usually observed after PFF addition in neurons. Could the authors explain, elaborate or validate their fibrils on primary neurons as well?

Antibody used for WB is not specific to human aSyn; therefore it's bit surprising that authors do not detect any endogenous aSyn in brain samples seen in fig 1. Please elaborate

aSyn monomer is not necessarily good control group in conducted experiments since in itself it can have effects on inflammatory cytokines etc. It would be desirable to have both sham/unijected control, and monomer groups, otherwise it's hard to asses what is basal level. The authors alleviate this by experiment in Fig S4. Actually this data might be even more desirable in the main text.

Because there were quite clear effects of monomer aSyn (even if lower than PFFs) on proinflammatory cytokines, it's unclear if observed effects are specific to aSyn, or would theiy be also induced by injection of other human recombinant protein. Please at least discuss this limitation.

The authors should not use "data not shown". Either show the data e.g. in supplement, or remove the statement altogether and reference to missing data altogether.

Inclusion of timeline with clear description of timing of each test relative to PFF injections would be beneficial.

High quality images in provided image file for Figure 5 can bezoomed into to see aSyn aggregates ,however very little can be seen on standard magnification. Please provide additional zoomed in images of specific aggregates so that it can be seen by readers of published manuscript.

The amount of observed aggregates is modest. How do the authors exclude possibility that with repeated injections of PFFs into the CSF some of PFFs actually were transferred into brain parenchyma when needle was traversing it ?.

Minor:

In the introduction the authors write Baak staging. Please also mention recent data regarding several possible patterns of spreading depending on origin of aSyn pathology (Brain-first / body-first) e.g.

Borghammer P, Van Den Berge N. Brain-First versus Gut-First Parkinson's Disease: A Hypothesis. J Parkinsons Dis. 2019;9(s2):S281-S295. doi: 10.3233/JPD-191721. PMID: 31498132; PMCID: PMC6839496.

Authors refer to Figure S6, probably meaning Figure S5.

## Author Response

Revisions round 2 Reviewer 1 The authors have made considerable work expanding background and discussion in the manuscript. The authors also satisfactorily addressed most of the methodological concerns. However, still some major points remain problematic: Comment 1: Seeding capacity of used PFFs remain unaddressed methodological concern - was not addressed due to technical problems. While we agree that authors provide multiple other lines of PFF validation, lack of validation of potency of their PFFs in primary neurons is still major shortcoming. Nonetheless, provided characterization together with observation of in vivo aggregates which do resemble what is typically seen in literature validate that PFFs were functional. Still, it's impossible to assess how potent they were in induction of pathology - i.e. whether it was comparable with other studies. This would require study on primary neurons since data from immortalized SH-SY5H cell line is not convincing. The authors should provide validation in primary cultures, or from in vivo - from animals injected into striatum. Or at the very least discuss the possible shortcomings of the lack of validation in primary cultures. To address this important concern, we set up a collaboration (lab of prof. De Groef, KU Leuven, Belgium) and validated the seeding capacity of our α-syn PFF on primary hippocampal neurons, whereby we based ourselves on the protocol described by Lee et al1 . We confirmed the seeding capacity of sonicated PFF by showing that these PFF, but not Mono, were able to convert endogenous α-syn into aggregated α-syn phosphorylated at Serine 129 (pS129 α-syn) in primary neurons (Rebuttal Figure 1 and revised manuscript Figure 1-1I). Rebuttal Figure 1. Representative confocal images of pS129 a-syn (red) in primary hippocampal neurons after 11 days of incubation with Mono or PFF (n=3). Cell nuclei are counterstained with Hoechst (blue) and neurons are counterstained with b-III-tubulin (green). Scale bar represents 20 µm. The following sentence was added to results line 427: "Sonicated PFF but not Mono were able to convert endogenous α-syn into aggregated α-syn phosphorylated at Serine 129 (pS129 α-syn) in primary hippocampal neurons (Figure 1-1I), again confirming their seeding capacity." The accompanying materials and methods section is added to materials and methods line 219-240. Comment 2: In the first round of revisions, the following comment was raised: The antibody used for WB is not specific to human aSyn; therefore it's bit surprising that authors do not detect any endogenous aSyn in brain samples seen in fig 1. Please elaborate. To this comment, we replied as follows: In Figure 1, we are analyzing the presence of α-syn in the CSF and several brain regions (i.e., ChP, OB and striatum) shortly after the icv injection (i.e., after 1 h, 8 h and 24 h). In all brain regions we can detect a clear α-syn signal 1h after injection, but not (clearly) anymore at later timepoints after PFF injection. This indeed indicates that we are not detecting endogenous α-syn which is expected to be present at each timepoint. Instead, we believe that we are visualizing the injected PFF which are present in higher concentrations - especially shortly (e.g., 1 h) after the icv injection - compared to endogenous α-syn. As such, we probably readily pick up the higher concentration of injected PFF whereas more sensitive settings may be needed to visualize endogenous α-syn." Now, the following comment was raised: Answer to this question further strengthens concerns about sensitivity of WB. If the authors can detect only very high levels of aSYN, then lack of signal in more distant structures is hard to interpret - there still might be enough exogenous aSYN there to induce pathology, while it's not detectable since it spread to larger brain volume, thus reducing concentration in particular structures. Our aim with the western blot shown in Figure 1 was comparing the distribution of PFF versus Mono at different timepoints post injection (i.e., after 1 h, 8 h and 24 h) within specific tissues (i.e., ChP, OB and striatum). Hereby, equal protein amounts were loaded per tissue (i.e., 20 µg for ChP and 80 µg for OB and striatum samples), allowing comparison between different timepoints. Furthermore, we concluded that comparing the three tissues, the highest signal was present 1 h after PFF injection in the ChP since four times less protein was loaded for this tissue (i.e. 20 µg) compared to the OB and striatum samples (i.e. 80 µg). However, given the remark of the reviewer, we agree that this claim may be confounded by the brain volume of each tissue. Therefore, we removed this sentence from the manuscript. We indeed agree with the reviewer that the α-syn PFF can be taken up by regions surrounding the ventricular system, including the striatum and the OB. This is also shown in our western blot, where we can detect a clear α-syn signal 1h after PFF injection in the OB and the striatum. We also believe that these PFF may initiate pathology in these regions. Consequently, the following sentence was already included in discussion line 653: We believe that the pS129 α-syn pattern we observe 2 months post-injection is mainly caused by seeding following uptake of α-syn PFF by brain regions surrounding the ventricular system and subarachnoid space. In addition, the limitation regarding sensitivity of the western blot data is now acknowledged in discussion line 585: Comparing the a-syn signal between different tissues may however be confounded by the size of the tissues (i.e,. more diluted a-syn signal in larger tissues), indicating that less detectable a-syn signal does not exclude pathological relevance. Revisions round 1 Reviewer 1 This study is focused on "The spreading and effects of human recombinant alpha-synuclein pre-formed fibrils in the cerebrospinal fluid of mice". The authors used in vivo methods to evaluate the impact of alpha-synuclein pre-formed fibrils when injected into the CFS of mice. They replicated data from the literature showing that PFF administration in the lateral ventricle of mice results in long-term PD-like pathology. The literature review in the introduction part does not give enough context for the reader to find the relevance of choroid plexus (ChP) in PD pathology. It is quite scarcely written and does not clearly state the study objectives and their novelty. Research gaps are not clearly emphasized in the introduction part of the manuscript, so they should be more clearly written. Future applications of the study results are missing. Therefore, it is hard to see the relevance of this study. The authors should highlight the importance and novelty of their study and its applicability in the future and medicine more clearly. The experiments appear to be well conducted. However, the results are rather descriptive, and real molecular interactions are not investigated. Finally, the manuscript completely lacks depth beyond describing that α-syn PFF infusion by CFS route is an appropriate model to study PD-like pathology. The relevance of the findings is very questionable. According to the reviewer, we replicated data from literature whereby the relevance and novelty of our findings is questionable. We do not agree with this comment, since in our manuscript we provide novel data by combining an intracerebroventricular (icv) injection model with well-characterized seeding competent alpha-synuclein (α-syn) pre-formed fibrils (PFF). Hereby, we specifically investigate the spreading of α-syn PFF throughout the cerebrospinal fluid (CSF) which has not been studied before. In contradiction with the opinion of the reviewer, we believe that our findings are of great relevance given accumulating data about the importance of the presence of α-syn seeds in CSF of human patients. We further elaborate on these comments below. Well-characterized seeding competent α-syn PFF are widely used as a preclinical model for Parkinson's disease (PD) upon injection into specific regions of the mouse brain or the enteric nervous system. In our study, to the best of our knowledge, we are the first to introduce these PFF into the CSF of wild type (WT) mice. A few other studies also applied an icv injection method in the context of PD, but in these studies less-well characterized a-syn oligomers2-5 or fibrils3 were used which affects the reproducibility of the data and renders the comparison with PFF-based brain injection models more difficult. Despite this, one study indeed described the occurrence of a PD-like phenotype consisting of olfactory dysfunction 4 days after injection and motor function deficits 45 days after injection2 , whereas another study reported acute but transient memory impairment in the novel object recognition test3,4, both after injection of a-syn oligomers. Importantly, we are the first to investigate the CSF-spreading of α-syn PFF and its short- and long-term consequences on the brain, which we believe is of major importance given the accumulating data about the presence of α-syn seeds in CSF of human patients. Of note, very recently it has been shown that α-syn seeds can already be detected in CSF of prodromal PD patients6 , underlining its pathological importance even early in the disease progression. These findings strengthen our belief that characterizing the spreading pattern of CSF α syn and identifying its consequences on the brain will increase our understanding of PD pathology, which may lead to novel strategies to impact α-syn spreading through the brain or attenuate brain deterioration in the future. We integrated these arguments in the revised manuscript: Line 5-21 (abstract): Parkinson's disease (PD) patients harbour seeding competent α-syn in their cerebrospinal fluid (CSF), which is mainly produced by the choroid plexus (ChP). Nonetheless, little is known about the role of the CSF and the ChP in PD pathogenesis. To address this question, we used an intracerebroventricular (icv) injection mouse model to assess CSF α-syn spreading and its short- and long-term consequences on the brain. Hereby, we made use of seeding competent, recombinant α-syn pre-formed fibrils (PFF) that are known to induce aggregation and subsequent spreading of endogenous α-syn in stereotactic tissue injection models. Here, we show that icv injected PFF, but not monomers (Mono), are rapidly removed from the CSF by interaction with the ChP. Additionally, shortly after icv injection both Mono and PFF were detected in the olfactory bulb and striatum. This spreading was associated with increased inflammation and complement activation in these tissues as well as leakage of the blood-CSF barrier. Despite these effects, a single icv injection of PFF didn't induce a decline in motor function. In contrast, daily icv injections over the course of five days resulted in deteriorated grip strength and formation of phosphorylated α-syn inclusions in the brain two months later, whereas dopaminergic neuron levels were not affected. These results point towards an important clearance function of the CSF and the ChP which could mediate removal of PFF from the brain, whereby chronic exposure to PFF in the CSF may negatively impact blood-CSF barrier functionality and PD pathology. Line 77-91 (introduction): "Ongoing pathological changes in the brain are known to be reflected in the cerebrospinal fluid (CSF), making this fluid that flows inside the brain ventricles and the subarachnoid space (SAS) an ideal source for diagnostic markers. Indeed, seed amplification assays (SAA) show the presence of misfolded a-syn seeds with self-aggregating properties in CSF samples from synucleinopathy patients whereas no seeding activity could be detected in non-synucleinopathy controls7-13. Strikingly, a large-scale cross sectional study showed that CSF a-syn SAA can differentiate PD patients from healthy controls with high sensitivity and specificity6 . Moreover, the assay can detect prodromal individuals before diagnosis, indicating that the presence of CSF a-syn seeds might be an early indicator of synucleinopathy6 . Despite the knowledge on the (early) presence of seeding-competent a-syn in the CSF, up until now the CSF spreading of PFF and the following consequences have not been investigated. To address this knowledge gap, we used an intracerebroventricular (icv) injection model to introduce α-syn PFF directly into the CSF of wild-type mice, followed by analysis of the α-syn spreading and its short- and long-term consequences on the brain." Line 526-537 (discussion): "Additionally, protein amplification assays indicate the presence of misfolded a-syn seeds with self aggregating properties in CSF samples from synucleinopathy patients8-12 and even in prodromal PD patients6 , which may underline the importance of CSF a-syn seeds even early on in PD pathology. Furthermore, a newly developed tool (i.e. Fluorescent Reporter for human a-syn or FluoReSyn) indicates that a-syn present in human CSF is able to enter into FluoReSyn reporter cells, pointing towards a potential role of the CSF in mediating a-syn transmission14. Together, these results clearly support the presence of a-syn aggregates harbouring seeding capacity in CSF of PD patients. Nevertheless, not much is known about the distribution of pathogenic a-syn species throughout the CSF and their effect on surrounding brain structures, even though these findings could lead to novel strategies to impact α syn spreading through the brain or attenuate brain deterioration in the future." To address another concern of the reviewer, namely that the literature review in the introduction part does not give enough context to find the relevance of the choroid plexus (ChP) in PD pathology, we specifically expanded this section of the introduction. Indeed, investigating the effect of CSF a-syn on the ChP represented a major focus of our study since the majority of CSF is secreted by the ChP, a highly vascularized structure hanging inside the brain ventricles. Furthermore, the ChP is a largely understudied brain region in PD despite its importance in the pathology of neurodegenerative diseases, as is evident from for example the Alzheimer's disease field. These notions were added to revised manuscript line 99-116: "It is well known that aging - the biggest risk factor for the development of PD - affects the morphology and functionality of the blood-CSF barrier leading to a compromised barrier integrity and reduced CSF turnover, amongst others15,16. Of note, alterations in CSF drainage and ChP transporter function are believed to contribute to Alzheimer's disease (AD) pathology by affecting the clearance of amyloid beta (Ab) 17, but it is currently unknown whether similar mechanisms apply to PD. Nonetheless, although little is known about the functionality of the blood-CSF barrier in PD, the available data point towards its potential importance. Indeed, a-syn immunoreactivity is present in human ChP tissue derived from patients with synucleinopathies including PD, although the signal is variable18. This suggests that the ChP might take up a-syn that is present in the CSF, thereby representing a potential clearance mechanism for a-syn removal from the brain. Transwell experiments using immortalized ChP epithelial cells showed that ChP epithelial cells are capable of taking up and transporting exogenously added a syn from the inner compartment (resembling the CSF side) to the outer compartment (resembling the blood side) and vice versa19. Additionally, an increased albumin CSF-to-serum ratio suggestive of an increased blood-CSF barrier leakage has been described in PD patients with advanced disease20, but not in early-phase PD patients20,21. Together, these data suggest that the ChP might play a role in clearing a-syn from the brain and that the ChP functionality could be affected during PD, similar to what is described for AD." Finally, as asked by the reviewer, we now described our specific research objective more clearly in the revised manuscript line 117-122: "Previously, it has been reported that the blood-CSF barrier is affected in mouse models of peripheral inflammation22-24, in Niemann-Pick type C (NPC) disease25 and in AD24,26-28. In the present study, we assessed the functionality of the blood-CSF barrier and the role of the CSF in the spreading of a-syn PFF - two understudied topics in the PD field - using an icv injection model." Methods are overall well described, figures are carefully prepared and body text is well written. However study design suffers from some issues regarding proper control groups, which at least have to be thoroughly discussed as limitation. Additionally, the authors have to clarify some other technical question before the manuscript could be considered for publication. Major comments 1. Validation of proper PFF formation is important, the authors have performed many steps to ensure formation of amyloid, templating aSyn fibrils, with proper size range. However this still have to be validated for ability to induce aggregation in cells. In supplementary figure S1I the authors demonstrate ability of prepared PFFs to cause aggregation in SY5Y cells -however observed pS129-aSyn formed rather fuzzy image rather than dense aggregates usually observed after PFF addition in neurons. Could the authors explain, elaborate or validate their fibrils on primary neurons as well? We agree that the pS129 α-syn inclusions that we observed in the SH-SY5Y cells look different than inclusions observed in primary neurons and in vivo. Therefore, as suggested by the reviewer, we aimed to also validate the seeding capacity of our α-syn PFF on primary hippocampal neurons, whereby we based ourselves on the protocol described by Lee et al1 . Unfortunately, despite several attempts, we were unable to obtain a successful neuronal culture, rendering us unable to proceed with PFF stimulation. Due to the extensive timeline required by this experiment (i.e., approximately 10 days of culturing neurons and approximately 14 days between stimulation with PFF and pS129 α-syn staining), we therefore could not confirm the seeding capacity of the α-syn PFF on primary neurons within the 3-month revision period. We however would like to highlight that we validated proper fibril formation and activity using multiple other readouts, in line with current recommendations in the field29. Indeed, we confirmed amyloid conformation using the Thioflavin T assay and aggregate formation using the sedimentation assay and electron microscopy prior to sonicating the PFF. Furthermore, post-sonication we validated the size of the fibrils using electron microscopy, their seeding capacity using the Thioflavin T assay and in vitro stimulation of SH-SY5Y neuroblastoma cells and their in vivo pathogenicity by formation of pS129 α syn inclusions in the brain 2 months post repeated icv injections. Although we do agree that evaluating the seeding capacity of PFF on primary neurons is preferred over seeding experiments in an immortalised cell line, we also believe that our in vivo experiment is the primary readout confirming the seeding capacity of our PFF, which we hope the reviewer can agree with. If not, we will look for alternatives to obtain primary neuronal cultures. 2. Antibody used for WB is not specific to human aSyn; therefore it's bit surprising that authors do not detect any endogenous aSyn in brain samples seen in fig 1. Please elaborate. In Figure 1, we are analyzing the presence of α-syn in the CSF and several brain regions (i.e., ChP, OB and striatum) shortly after the icv injection (i.e., after 1h, 8h and 24h). In all brain regions we can detect a clear α-syn signal 1h after injection, but not (clearly) anymore at later timepoints after PFF injection. This indeed indicates that we are not detecting endogenous α-syn which is expected to be present at each timepoint. Instead, we believe that we are visualizing the injected PFF which are present in higher concentrations - especially shortly (e.g., 1 h) after the icv injection - compared to endogenous α-syn. As such, we probably readily pick up the higher concentration of injected PFF whereas more sensitive settings may be needed to visualize endogenous α-syn. 3. aSyn monomer is not necessarily good control group in conducted experiments since in itself it can have effects on inflammatory cytokines etc. It would be desirable to have both sham/uninjected control, and monomer groups, otherwise it's hard to asses what is basal level. The authors alleviate this by experiment in Fig S4. Actually this data might be even more desirable in the main text. Because there were quite clear effects of monomer aSyn (even if lower than PFFs) on proinflammatory cytokines, it's unclear if observed effects are specific to aSyn, or would they be also induced by injection of other human recombinant protein. Please at least discuss this limitation. Injecting a substance into the brain ventricles can already induce some effects. Therefore, in the experiments where we investigated short term effects, we included two different control groups. One group of control mice was injected with the same amount of Mono (i.e. 5 µl of 1 µg/µl), the starting material from which PFF are generated and therefore the most recommended control protein according to the general guidelines in the field29. Additionally, another group of mice received the same volume (i.e., 5 µl) of PBS - the solvent used to dilute PFF and Mono - and therefore represents a control for the icv injection procedure itself. We believe that the comparison of PBS, Mono and PFF injected mice represents an important strength of our study since most published studies only include one control condition. We indeed observed that Mono icv injection induced more pronounced effects on several readouts, including blood-CSF barrier permeability and inflammatory response, in comparison with icv PBS injection. Nonetheless, it may not be unexpected that a sudden increase in the concentration of Mono in the CSF also induces a brain response and importantly, the PFF effect was consistently more pronounced. The latter indicates that the conformation of the injected α-syn species (i.e., Mono versus PFF), and not the injection of an equal amount of α-syn protein per se, determines the inflammatory response. For the long-term analysis of the PFF icv injection mouse model, we only included one control group to facilitate the feasibility of the experiment. Hereby, we chose a PBS control group because it has been reported that the instrastriatal injection of Mono in rats seeded a small number of pS129 α-syn positive aggregates in the substantia nigra pars compacta 6 months after injection, which might have been due to low levels of spontaneous aggregates in the Mono30. We have extensive expertise with icv injections and also implemented this approach as an acute model for Alzheimer's disease by injecting amyloid beta oligomers (AβO) or a scrambled control peptide in CSF of wild-type mice26-28. Indeed, also here we could observe several AβO-induced effects on the ChP, including leakage of the blood-CSF barrier and inflammatory gene expression. Importantly, these AβO induced effects occurred in comparison to a scrambled peptide control condition which has the same amino acid sequence as Aβ1-42, but in a random order. Therefore, this scrambled peptide is not expected to assemble into oligomers but does allow to control for both the icv injection procedure and the introduction of a certain concentration of a foreign protein into the CSF. In conclusion, although not any protein that is introduced into the CSF will impact the ChP to the same extent, different disease-specific proteins known to be present in the CSF of patients (e.g., AβO in case of AD patients or α-syn in case of PD patients) can cause similar effects. We added the following paragraph to revised manuscript line 600-610 (discussion): "This suggests that the elicited pro-inflammatory response is induced by a-syn itself and that PFF harbour a higher pro-inflammatory capacity in comparison with Mono. Of note, also Mono induced a higher inflammatory gene expression in comparison with mice injected with PBS, a control group for the icv injection procedure itself, although the PFF effect was in general the most pronounced. Nonetheless, we can't pinpoint the specificity of this response to the icv injection of PFF since we have previously shown that also the icv injection of AβO affects the functionality of the blood-CSF barrier26- 28. However, our data do allow us to conclude that not any protein that is introduced into the CSF will impact the ChP to the same extent, but that different disease-specific proteins known to be present in the CSF of patients (e.g., AβO in case of AD patients or α-syn in case of PD patients) may cause similar effects." 4. The authors should not use "data not shown". Either show the data e.g. in supplement, or remove the statement altogether and reference to missing data altogether. Since the missing data did not substantially add to the results described in the manuscript, the statements were removed from the manuscript. 5. Comments regarding Figure 5 a. Inclusion of timeline with clear description of timing of each test relative to PFF injections would be beneficial. A timeline was included in revised manuscript Figure 5-2 (rebuttal Figure 1). Rebuttal Figure 1. Overview of experimental setup of repeated intracerebroventricular (icv) injection experiment. At baseline (day 0), mice were first assessed using the beam traversal test, followed by the pole descent test and finally the inverted grid test. Training for the beam traversal test and pole descent test was performed on the days preceding day 0 following the schedule as described in the materials and methods section. From day 1 until day 5, mice were icv injected daily with either 5 µl of 1 μg/μl human alpha synuclein (α-syn) pre-formed fibrils (PFF) or an equal volume of PBS. Approximately 2 months later, on day 77, mice were again assessed using the beam traversal test, followed by the pole descent test and finally the inverted grid test. Training for the beam traversal and pole descent test was not repeated at this timepoint. On day 80, mice were sacrificed. b. High quality images in provided image file for Figure 5 can be zoomed into to see aSyn aggregates, however very little can be seen on standard magnification. Please provide additional zoomed in images of specific aggregates so that it can be seen by readers of published manuscript. To address this comment, we added a representative, digital zoom-in on revised manuscript Figure 5B (rebuttal Figure 2). This zoom-in clearly shows the presence of fibrillar pS129 α-syn aggregates, similar to globular Lewy bodies and thread-like Lewy neurites as seen in humans. Rebuttal Figure 2. Representative images of α-syn phosphorylated at Serine 129 (pS129 α-syn; brown) in the brain 2 months after the icv injection of pre-formed fibrils (PFF). Scale bar represents 100 μm. c. The amount of observed aggregates is modest. How do the authors exclude possibility that with repeated injections of PFFs into the CSF some of PFFs actually were transferred into brain parenchyma when needle was traversing it? We indeed can't exclude that small amounts of α-syn seeds were deposited along the injection needle tract. However, like most PFF studies in literature, we made use of a 10 µl Hamilton syringe combined with a slow injection rate and a waiting time after injection with the needle in place to minimize this risk. Two months after our repetitive injection scheme (i.e., daily icv injection over the course of 5 days), we observed pS129 α-syn inclusions in the cortex and hippocampus with clear cortical inclusion in both hemispheres. However, we did not see clear pS129 α-syn inclusions in the striatum or substantia nigra. If we suppose that α-syn PFF deposits were left in our tract towards the lateral ventricle, these would have ended up in the cortex (cfr. injection coordinates relative to bregma: AP - 0.7 mm, ML + 1.0 mm, DV -2.0 mm). Nonetheless, the pS129 α-syn distribution pattern we observe does not correlate with the pattern described by the few studies that used cortical PFF injection as a primary location of injection. Indeed, in the first seminal paper of Luk et al. which targeted the cortex (AP +0.2 mm, ML +2.0 mm, DV -0.8 mm), pS129 α-syn inclusions were seen in the frontal cortex, striatum and amygdala at 180 days post-injection31. It was not described if the contralateral hemisphere also showed pS129 α-syn pathology, but no pS129 α-syn inclusions were seen at the hippocampus or substantia nigra31. In the study of Osterberg et al., unilateral injection localized to the sensory cortex (AP +0.0 mm, ML +1.0 mm, DV -1.2 mm) produced a clear spread of aggregation to the contralateral sensory cortex 3 to 4 months post-injection32. This spread to the contralateral region was not described in the sampling 2 months post-injection, contradictory to what we observed in our repetitive icv injection model. In conclusion, the location of the pS129 α-syn inclusions in our model is hard to explain through propagation of pathology between brain regions connected via neuronal pathways, which has been proven to be the main propagation method in most intracerebral tissue injections33. Therefore, we believe that the pS129 α-syn pattern we observe 2 months post-injection is mainly caused by seeding following uptake of α-syn PFF by brain regions surrounding the ventricular system and subarachnoid space. We integrated these arguments in the revised manuscript line 639-653: "In the long-term, a single icv injection of human PFF was unable to induce deficits in motor function. This could be the result of clearance of PFF from the CSF, thereby precluding seeding of a-syn pathology. This hypothesis is reinforced by our follow-up experiment, where maintaining a higher PFF concentration over a prolonged time course resulted in a deterioration in grip strength and pS129 α syn deposition in the brain 2 months post injection although dopaminergic neurons were not affected. In patients, a chronic exposure to seeding competent α-syn over the long disease process might eventually lead to similar results. Of note, in our icv injection paradigm we can't exclude that small amounts of α-syn seeds were deposited in the cortex along the injection needle tract. However, like most PFF studies in literature, we made use of a 10 µl Hamilton syringe combined with a slow injection rate and a waiting time after injection with the needle in place to minimize this risk. Furthermore, the pS129 α-syn distribution pattern we observe does not correlate with the pattern described by the few studies that used cortical PFF injection as a primary location of injection31,32. Therefore, we believe that the pS129 α-syn pattern we observe 2 months post-injection is mainly caused by seeding following uptake of α-syn PFF by brain regions surrounding the ventricular system and subarachnoid space." Minor comments 1. In the introduction the authors write Baak staging. Please also mention recent data regarding several possible patterns of spreading depending on origin of aSyn pathology (Brain-first / body-first) e.g. Borghammer P, Van Den Berge N. Brain-First versus Gut-First Parkinson's Disease: A Hypothesis. J Parkinsons Dis. 2019;9(s2):S281-S295. doi: 10.3233/JPD-191721. PMID: 31498132; PMCID: PMC6839496. The following paragraph was added to revised manuscript line 61-66: "More recently, it has been hypothesized that PD comprises a brain-first and a body-first subtype consisting of different spreading patterns. Hereby, it is proposed that the initial α-syn pathology appears in the central nervous system (more specifically the amygdala or connected structures and in some cases the OB) before descending to the peripheral nervous system whereas the reversed pattern is described in the body-first subtype (i.e., peripheral nervous system prior to central nervous system)34." 2. Authors refer to Figure S6, probably meaning Figure S5. According to this comment, references to Figure S6 (now Figure 5-1) were adapted in revised manuscript line 484-486. References 1 Volpicelli-Daley, L. A. et al. Addition of exogenous α-synuclein preformed fibrils to primary neuronal cultures to seed recruitment of endogenous α-synuclein to Lewy body and Lewy neurite-like aggregates. Nature Protocols 9, 2135, doi:10.1038/nprot.2014.143 PMID - 25122523 (2014). 2 Fortuna, J. T. S. et al. Brain infusion of α-synuclein oligomers induces motor and non-motor Parkinson's disease-like symptoms in mice. Behavioural brain research 333, 150-160, doi:10.1016/j.bbr.2017.06.047 PMID - 28668282 (2017). 3 Vitola, P. L. et al. Alpha-synuclein oligomers impair memory through glial cell activation and via Toll-like receptor 2. Brain Behav Immun 69, 591-602, doi:10.1016/j.bbi.2018.02.012 PMID - 29458199 (2018). 4 Vitola, P. L. et al. Cellular prion protein neither binds to alpha-synuclein oligomers nor mediates their detrimental effects. Brain 142, 249-254, doi:10.1093/brain/awy318 PMID - 30601948 (2019). 5 Vitola, P. L. et al. Peripheral inflammation exacerbates α-synuclein toxicity and neuropathology in Parkinson's models. Neuropath Appl Neuro, doi:10.1111/nan.12644 (2020). 6 Siderowf, A. et al. Assessment of heterogeneity among participants in the Parkinson's Progression Markers Initiative cohort using α-synuclein seed amplification: a cross-sectional study. Lancet Neurology 22, 407-417, doi:10.1016/s1474-4422(23)00109-6 PMID - 37059509 (2023). 7 Paciotti, S. et al. Are We Ready for Detecting α-Synuclein Prone to Aggregation in Patients? The Case of "Protein-Misfolding Cyclic Amplification" and "Real-Time Quaking-Induced Conversion" as Diagnostic Tools. Front Neurol 9, 415, doi:10.3389/fneur.2018.00415 PMID - 29928254 (2018). 8 Fairfoul, G. et al. Alpha-synuclein RT-QuIC in the CSF of patients with alpha-synucleinopathies. Ann Clin Transl Neur 3, 812-818, doi:10.1002/acn3.338 PMID - 27752516 (2016). 9 Shahnawaz, M. et al. Development of a Biochemical Diagnosis of Parkinson Disease by Detection of α Synuclein Misfolded Aggregates in Cerebrospinal Fluid. Jama Neurol 74, 163, doi:10.1001/jamaneurol.2016.4547 PMID - 27918765 (2016). 10 Groveman, B. R. et al. Rapid and ultra-sensitive quantitation of disease-associated α-synuclein seeds in brain and cerebrospinal fluid by αSyn RT-QuIC. Acta Neuropathologica Commun 6, 7, doi:10.1186/s40478-018-0508-2 PMID - 29422107 (2018). 11 Rumund, A. v. et al. α-Synuclein real-time quaking-induced conversion in the cerebrospinal fluid of uncertain cases of parkinsonism. Ann Neurol 85, 777-781, doi:10.1002/ana.25447 PMID - 30801759 (2019). 12 Rossi, M. et al. Ultrasensitive RT-QuIC assay with high sensitivity and specificity for Lewy body associated synucleinopathies. Acta Neuropathologica 140, 49-62, doi:10.1007/s00401-020-02160-8 PMID - 32342188 (2020). 13 Russo, M. J. et al. High diagnostic performance of independent alpha-synuclein seed amplification assays for detection of early Parkinson's disease. Acta Neuropathologica Commun 9, 179, doi:10.1186/s40478-021-01282-8 PMID - 34742348 (2021). 14 Gerdes, C. et al. A nanobody-based fluorescent reporter reveals human α-synuclein in the cell cytosol. Nat Commun 11, 2729, doi:10.1038/s41467-020-16575-0 PMID - 32483166 (2020). 15 Gorlé, N. et al. The effect of aging on brain barriers and the consequences for Alzheimer's disease development. Mamm Genome 27, 407-420, doi:10.1007/s00335-016-9637-8 PMID - 27143113 (2016). 16 Vandenbroucke, R. E. A Hidden Epithelial Barrier in the Brain with a Central Role in Regulating Brain Homeostasis. Implications for Aging. Annals of the American Thoracic Society 13, S407-S410, doi:10.1513/AnnalsATS.201609-676AW PMID - 28005425 (2016). 17 Serot, J.-M. et al. A Possible Role for CSF Turnover and Choroid Plexus in the Pathogenesis of Late Onset Alzheimer's Disease. J Alzheimer's Dis 30, 17-26, doi:10.3233/jad-2012-111964 PMID - 22349684 (2012). 18 Mollenhauer, B. et al. α-Synuclein in human cerebrospinal fluid is principally derived from neurons of the central nervous system. J Neural Transm 119, 739-746, doi:10.1007/s00702-012-0784-0 PMID - 22426833 (2012). 19 Bates, C. A. et al. Expression and Transport of a-Synuclein at the Blood-Cerebrospinal Fluid Barrier and Effects of Manganese Exposure. ADMET & DMPK 3, 15-33, doi:10.5599/admet.3.1.159 PMID - 26640596 (2015). 20 Pisani, V. et al. Increased blood-cerebrospinal fluid transfer of albumin in advanced Parkinson's disease. Journal of Neuroinflammation 9, 188, doi:10.1186/1742-2094-9-188 PMID - 22870899 (2012). 21 Haussermann, P. et al. Integrity of the blood-cerebrospinal fluid barrier in early Parkinson's disease. Neuroscience Letters 300, 182-184, doi:10.1016/s0304-3940(01)01574-9 PMID - 11226641 (2001). 22 Balusu, S. et al. Identification of a novel mechanism of blood-brain communication during peripheral inflammation via choroid plexus-derived extracellular vesicles. Embo Mol Med 8, 1162-1183, doi:10.15252/emmm.201606271 PMID - 27596437 (2016). 23 Gorlé, N. et al. The choroid plexus epithelium as a novel player in the stomach-brain axis during Helicobacter infection. (2018). 24 Xie, J. et al. Low-grade peripheral inflammation affects brain pathology in the AppNL-G-Fmouse model of Alzheimer's disease. Acta Neuropathologica Commun 9, 163, doi:10.1186/s40478-021-01253-z PMID - 34620254 (2021). 25 Hoecke, L. V. et al. Involvement of the Choroid Plexus in the Pathogenesis of Niemann-Pick Disease Type C. Front Cell Neurosci 15, 757482, doi:10.3389/fncel.2021.757482 PMID - 34720883 (2021). 26 Brkic, M. et al. Amyloid β Oligomers Disrupt Blood-CSF Barrier Integrity by Activating Matrix Metalloproteinases. The Journal of Neuroscience 35, 12766-12778, doi:10.1523/JNEUROSCI.0006- 15.2015 PMID - 26377465 (2015). 27 Steeland, S. et al. Counteracting the effects of TNF receptor-1 has therapeutic potential in Alzheimer's disease. Embo Mol Med 10, e8300, doi:10.15252/emmm.201708300 PMID - 29472246 (2018). 28 Vandendriessche, C. et al. Importance of extracellular vesicle secretion at the blood-cerebrospinal fluid interface in the pathogenesis of Alzheimer's disease. Acta Neuropathologica Commun 9, 143, doi:10.1186/s40478-021-01245-z PMID - 34425919 (2021). 29 Polinski, N. K. et al. Best Practices for Generating and Using Alpha-Synuclein Pre-Formed Fibrils to Model Parkinson's Disease in Rodents. J Park Dis 8, 303-322, doi:10.3233/jpd-171248 PMID - 29400668 (2018). 30 Paumier, K. L. et al. Intrastriatal injection of pre-formed mouse α-synuclein fibrils into rats triggers α synuclein pathology and bilateral nigrostriatal degeneration. Neurobiol Dis 82, 185-199, doi:10.1016/j.nbd.2015.06.003 PMID - 26093169 (2015). 31 Luk, K. C. et al. Pathological α-synuclein transmission initiates Parkinson-like neurodegeneration in nontransgenic mice. Sci New York N Y 338, 949-953, doi:10.1126/science.1227157 PMID - 23161999 (2012). 32 Osterberg, Valerie R. et al. Progressive Aggregation of Alpha-Synuclein and Selective Degeneration of Lewy Inclusion-Bearing Neurons in a Mouse Model of Parkinsonism. Cell Reports 10, 1252-1260, doi:10.1016/j.celrep.2015.01.060 PMID - 25732816 (2015). 33 Mezias, C. et al. Neural connectivity predicts spreading of alpha-synuclein pathology in fibril-injected mouse models: Involvement of retrograde and anterograde axonal propagation. Neurobiol Dis 134, 104623, doi:10.1016/j.nbd.2019.104623 PMID - 31628991 (2020). 34 Horsager, J. et al. Brain-first versus body-first Parkinson's disease: a multimodal imaging case-control study. Brain 143, 3077-3088, doi:10.1093/brain/awaa238 PMID - 32830221 (2020)
