# Supplementary figures and images for: The Spreading and Effects of Human Recombinant α-Synuclein Preformed Fibrils in the Cerebrospinal Fluid of Mice
Source: eNeuro. 2024 Mar 1;11(3):ENEURO.0024-23.2024. doi: 10.1523/ENEURO.0024-23.2024 (PMC10925901; doi:10.1523/ENEURO.0024-23.2024)

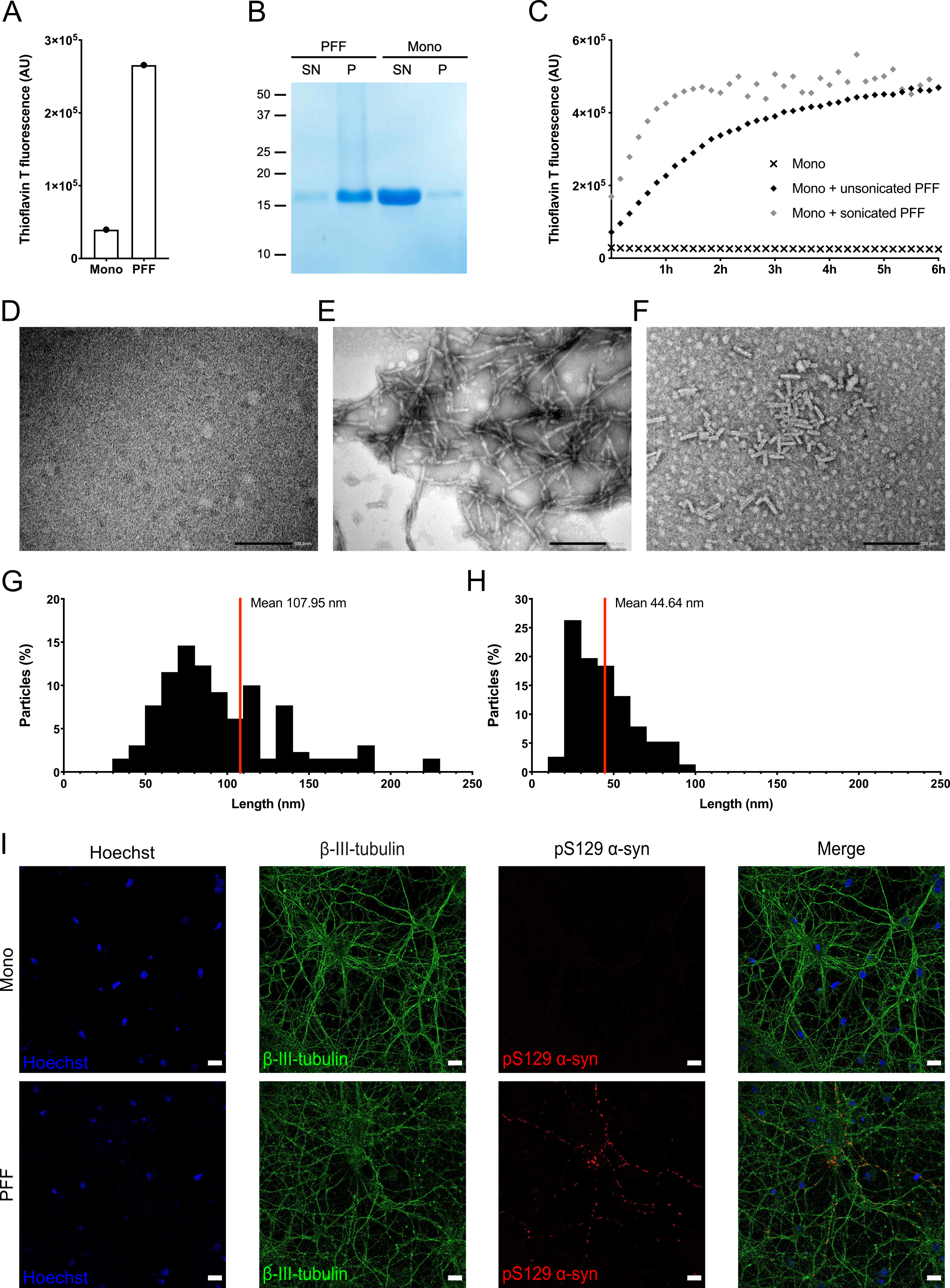

Supplement: Figure 1-1 — Characterization of human alpha synuclein (α-syn) pre-formed fibrils (PFF). Thioflavin T (ThT) assay of 0.05 mg/ml monomers (Mono) or PFF to confirm the presence of typical β-sheet structures in PFF but not in Mono. (B) Denaturing sodium dodecyl sulfate (SDS) polyacrylamide gel electrophoresis and Coomassie stain for volumetric analysis of the supernatant fraction (SN) and pellet fraction (P) of PFF and Mono, post-centrifugation. (C) ThT kinetic seeding assay to investigate recruitment of Mono by PFF. Seeds (unsonicated or sonicated PFF) (1 mg/ml) were added at a ratio of 1:20 to Mono (0.05 mg/ml). (D-F) Representative transmission electron microscopy (TEM) images of Mono (D), unsonicated PFF (E) and sonicated PFF (F). Scale bar represents 100 nm (D) or 200 nm (E, F). (G-H) Quantification of unsonicated (G) and sonicated (H) PFF fibril length measured from the representative TEM images. (I) Representative confocal images of pS129 α-syn (red) in primary hippocampal neurons after 11 days of incubation with Mono or PFF (n=3). Cell nuclei are counterstained with Hoechst (blue) and neurons are counterstained with β-III-tubulin (green). Scale bar represents 20 µm. Download Figure 1-1, TIF file. [file eneuro-11-ENEURO.0024-23.2024-s002.tif]

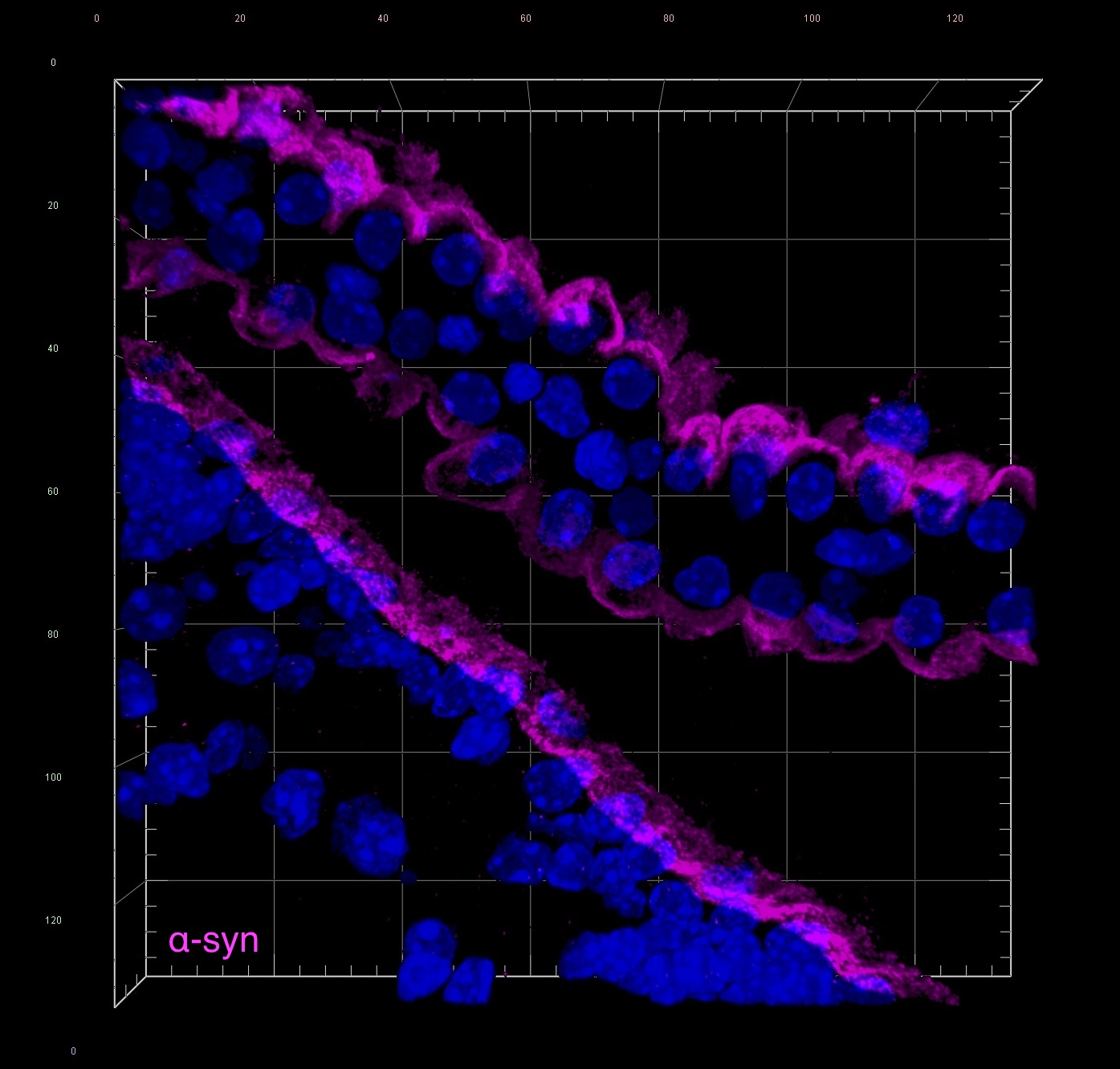

Supplement: Figure 2-1 — Analysis of the intraventricular localization of alpha-synuclein (α-syn) 1 h after the intracerebroventricular (icv) injection of human alpha synuclein (α-syn) pre-formed fibrils (PFF). Z-stack reconstruction of α-syn (magenta) in the choroid plexus (ChP) of the lateral ventricle 1 h after the icv injection of PFF (n=3). Cell nuclei are counterstained with Hoechst (blue). Scale bar (x-, y-, z-axis) represents 20 µm per square. Download Figure 2-1, TIF file. [file eneuro-11-ENEURO.0024-23.2024-s003.tif]

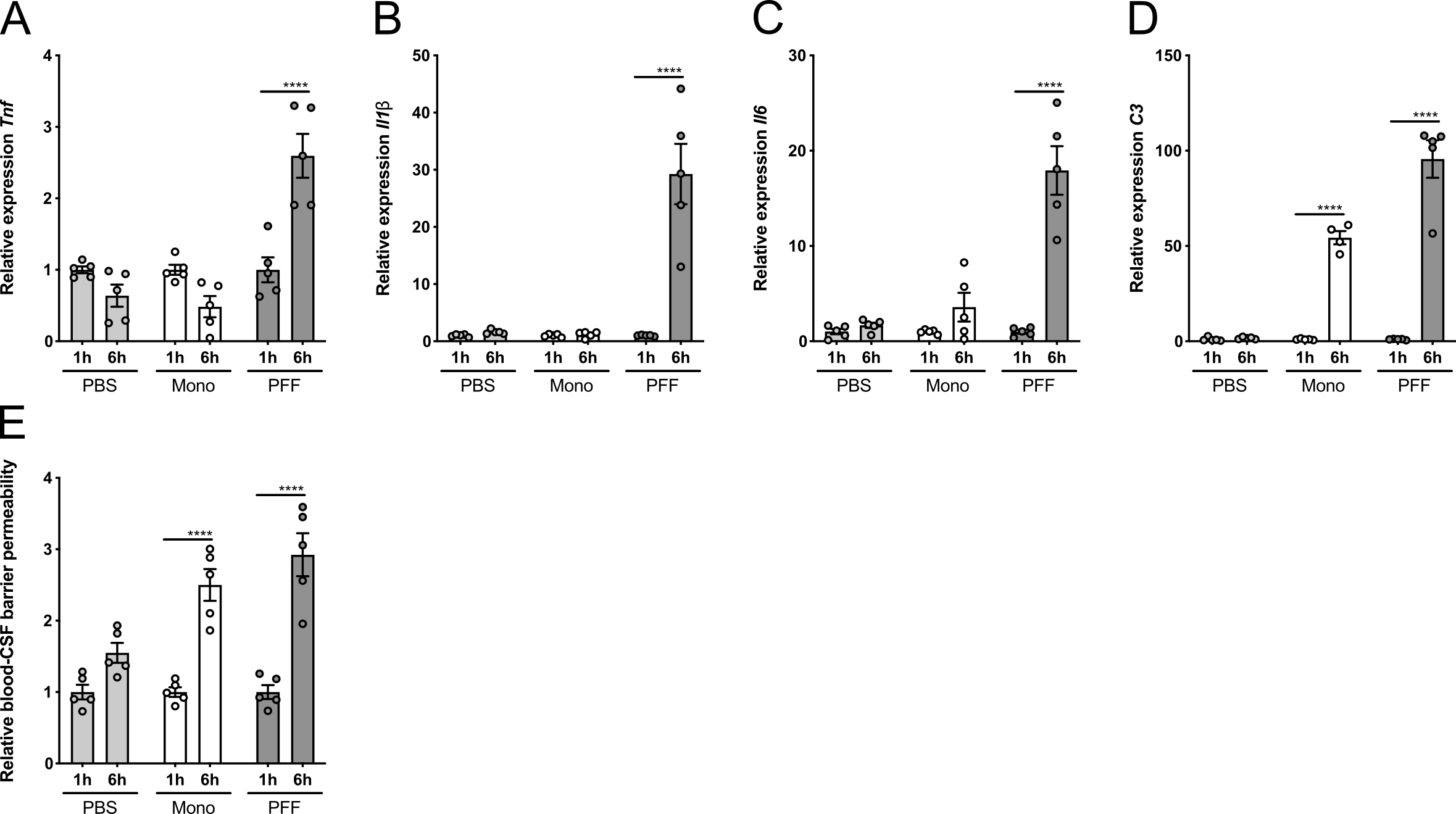

Supplement: Figure 3-1 — Comparison of the effects induced by human alpha synuclein (α-syn) pre-formed fibrils (PFF), monomers (Mono) and PBS 1h and 6 h after their intracerebroventricular (icv) injection. (A-D) qRT-PCR analysis of Tnf (A), Il1β (B), Il6 (C) and C3 (D) in the choroid plexus (ChP) 1 h and 6 h after the icv injection of PBS (light grey), Mono (black) or PFF (grey). Data are represented relative to the 1 h timepoint for each treatment condition (n=5). (E) Blood-CSF barrier permeability 1 h and 6 h after the icv injection of PBS (light grey), Mono (black) or PFF (grey) determined by intravenous (iv) injection of fluorescently labelled 4 kDa dextran followed by analysis of the fluorescence in the CSF. Data are represented relative to the 1 h timepoint for each treatment condition (n=5). Download Figure 3-1, TIF file. [file eneuro-11-ENEURO.0024-23.2024-s004.tif]

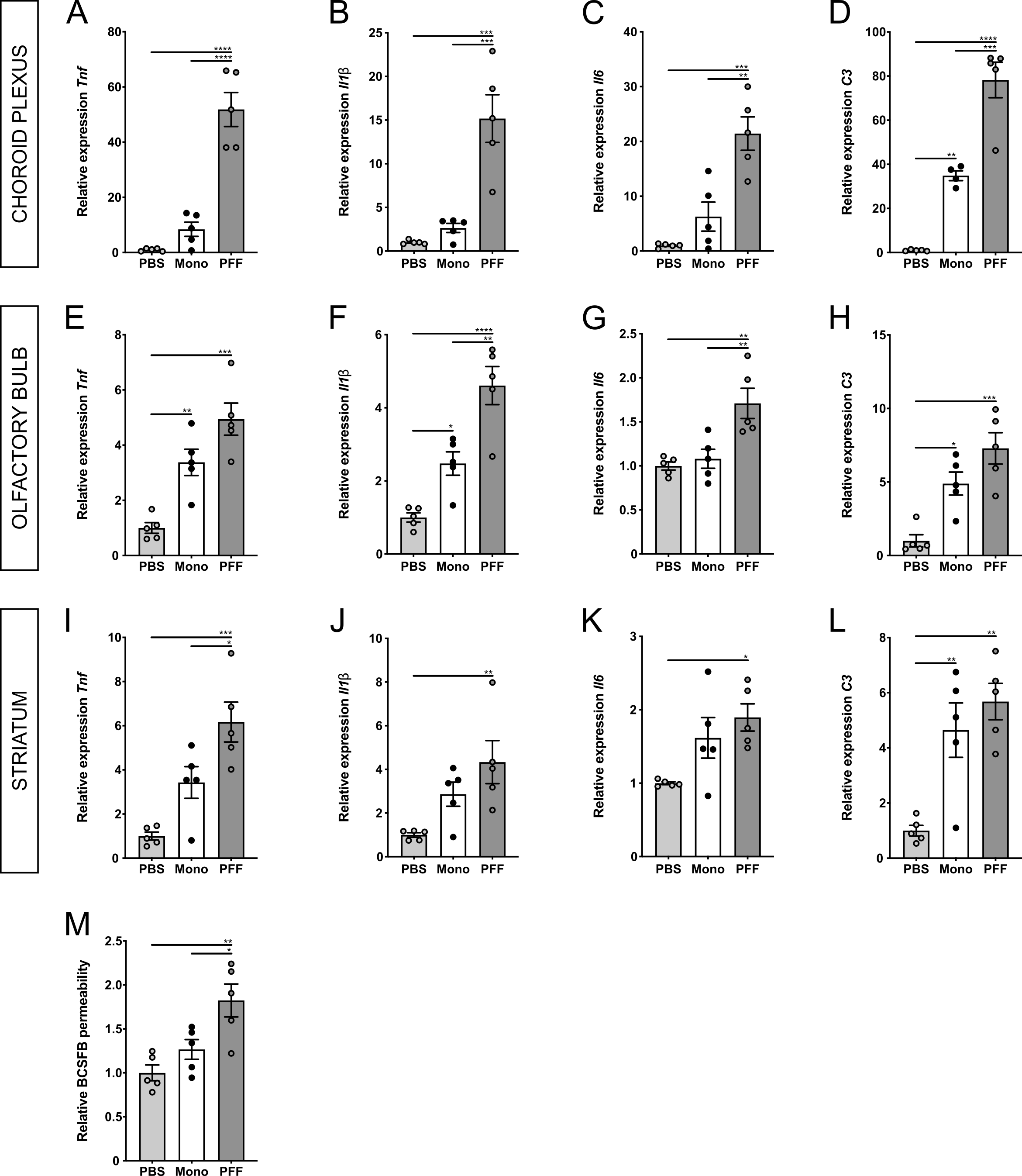

Supplement: Figure 3-2 — Comparison of the effects induced by human alpha synuclein (α-syn) pre-formed fibrils (PFF), monomers (Mono) and PBS 6 h after their intracerebroventricular (icv) injection. (A-D) qRT-PCR analysis of Tnf (A), Il1β (B), Il6 (C) and C3 (D) in the choroid plexus (ChP) 6 h after the icv injection of PBS (light grey), Mono (black) or PFF (grey). Data are represented relative to the PBS condition (n=5). (E-H) qRT-PCR analysis of Tnf (E), Il1β (F), Il6 (G) and C3 (H) in the olfactory bulb (OB) 6 h after the icv injection of PBS (light grey), Mono (black) or PFF (grey). Data are represented relative to the PBS condition (n=5). (I-L) qRT-PCR analysis of Tnf (I), Il1β (J), Il6 (K) and C3 (L) in the striatum 6 h after the icv injection of PBS (light grey), Mono (black) or PFF (grey). Data are represented relative to the PBS condition (n=5). (M) Blood-CSF barrier permeability 6 h after the icv injection of PBS (light grey), Mono (black) or PFF (grey) determined by intravenous (iv) injection of fluorescently labelled 4 kDa dextran followed by analysis of the fluorescence in the CSF. Data are represented relative to the PBS condition (n=5). Download Figure 3-2, TIF file. [file eneuro-11-ENEURO.0024-23.2024-s005.tif]

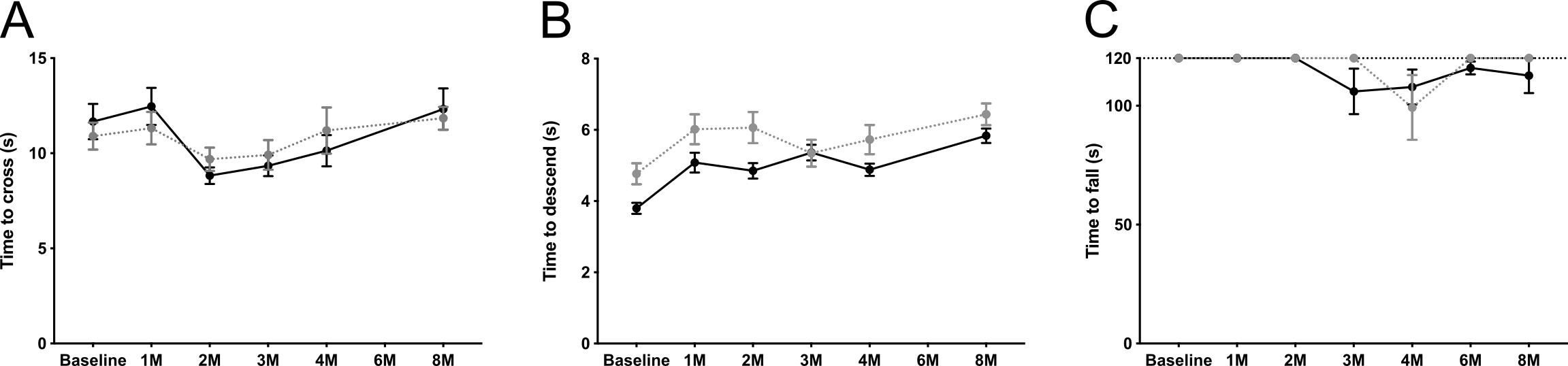

Supplement: Figure 5-1 — Analysis of motor function after the intracerebroventricular (icv) injection of human alpha synuclein (α-syn) pre-formed fibrils (PFF) and PBS. (A-C) Beam traversal (A), pole descent (B) and inverted grid (C) test at several timepoints after the icv injection of human PFF (grey) or PBS (black) (n=8). Download Figure 5-1, TIF file. [file eneuro-11-ENEURO.0024-23.2024-s006.tif]

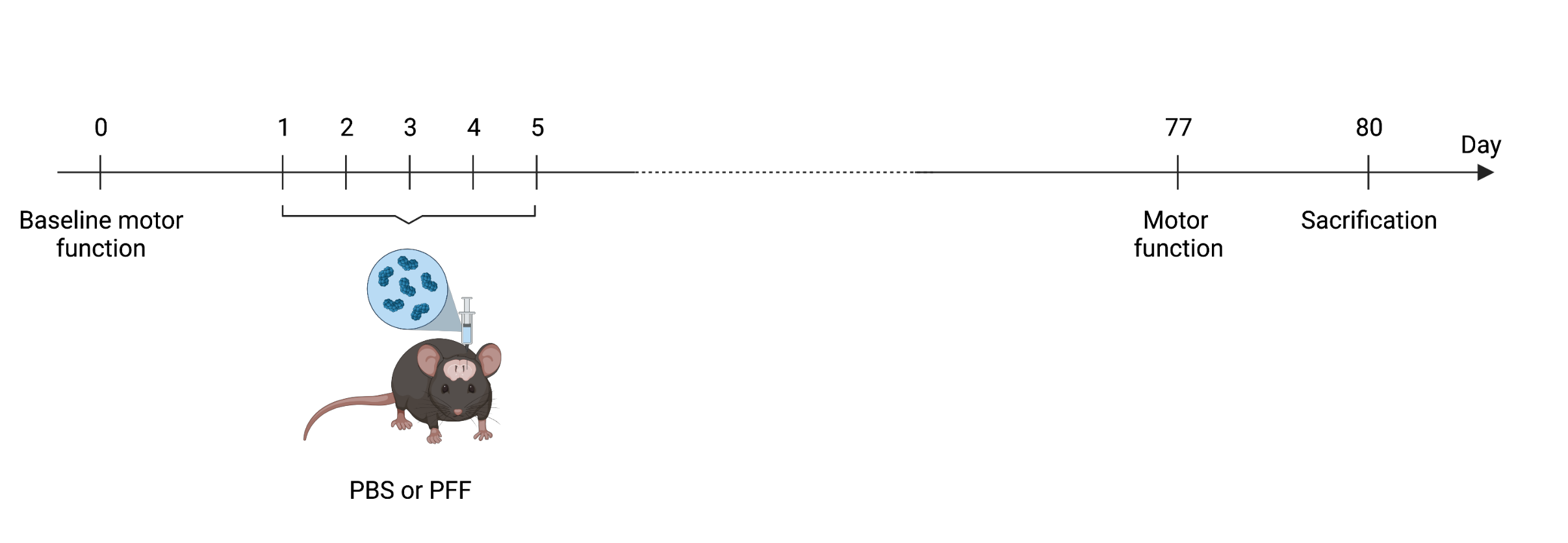

Supplement: Figure 5-2 — Overview of experimental setup of repeated intracerebroventricular (icv) injection experiment. At baseline (day 0), mice were first assessed using the beam traversal test, followed by the pole descent test and finally the inverted grid test. Training for the beam traversal test and pole descent test was performed on the days preceding day 0 following the schedule as described in the materials and methods section. From day 1 until day 5, mice were icv injected daily with either 5 µl of 1 μg/μl human alpha synuclein (α-syn) pre-formed fibrils (PFF) or an equal volume of PBS. Approximately 2 months later, on day 77, mice were again assessed using the beam traversal test, followed by the pole descent test and finally the inverted grid test. Training for the beam traversal and pole descent test was not repeated at this timepoint. On day 80, mice were sacrificed. Download Figure 5-2, TIF file. [file eneuro-11-ENEURO.0024-23.2024-s007.tif]
